# Genetic Mapping Reveals Broader Role of *Vrn-H3* Gene in Root and Shoot Development beyond Heading in Barley

**DOI:** 10.1371/journal.pone.0158718

**Published:** 2016-07-21

**Authors:** Md. Arifuzzaman, Süleyman Günal, Annemarie Bungartz, Shumaila Muzammil, Nazanin P. Afsharyan, Jens Léon, Ali Ahmad Naz

**Affiliations:** University of Bonn, Institute of Crop Science and Resource Conservation, Crop Genetics and Biotechnology Unit, Katzenburgweg 5, 53115, Bonn, Germany; Università Politecnica delle Marche, ITALY

## Abstract

The aim of the present study was to dissect the genetic inheritance and interplay of root, shoot and heading attributes for a better understanding of these traits in crop production. For this, we utilized quantitative trait loci (QTL) and candidate gene analysis approach using a second filial (F2) population originated from a cross between spring cultivar Cheri and wild barley accession ICB181160. The F2 population comprising 182 plants was phenotyped for root dry weight (RDW), root volume (RV), root length (RL) and shoot dry weight (SDW), tiller number per plant (TIL) and days to heading (HEA). In parallel, this population was genotyped using polymerase chain reaction (PCR) based cleaved amplified polymorphic sequence (CAPS) markers distributed across the whole genome. Marker by trait analysis revealed 16 QTL for root and shoot traits localized on chromosomes 1H, 3H, 4H, 5H and 7H. The strongest and a common QTL effect for root, shoot and heading traits was identified on chromosome 7H at the putative region of *Vrn-H3* gene. Later, we have established PCR based gene specific marker HvVrnH3 revealing polymorphism for early heading *Vrn-H3* allele in Cheri and late heading allele *vrn-H3* in ICB181160. Genotyping of these alleles revealed a clear co-segregation of early heading *Vrn-H3* allele with lower root and shoot attributes, while late heading *vrn-H3* allele with more TIL and higher root biomass suggesting a primary insight on the function of *Vrn-H3* gene beyond flowering. Genetic interactions of vernalization genes *Vrn-H3* with *Vrn-H2* and *Vrn-H1* also suggested the major role of *Vrn-H3* alleles in determining root and shoot trait variations in barley. We believe, these data provide an opportunity for further research to test a precise significance of early heading on yield components and root associated sustainability in crops like barley and wheat.

## Introduction

Land plants show polar development by which root and shoot are established during ontogeny. The root is the primary organ for water and nutrients absorption from soil and shoots consume these resources to convert them in agricultural production. The success of such relationship in crop production depends on excellent root-shoot attributes and their effective communications [1]. Therefore, genetic dissection of root and shoot traits and their putative association to reinforce plant performance is essential for the determination of favorable traits and to utilize the potentials of these parameters in crop production.

The members of dicotyledonous crops reveal a tap root system which differs from fibrous roots in monocotyledonous crops like wheat and barley. A major reason behind the fibrous root system in barley is the initiation of successive tillers during vegetative phase of plant development. These tillers produce nodal (adventitious) roots which penetrate easily in soil along with seminal roots and establish a network of extensive roots by producing lateral roots [2–3]. It was found that an increase in tiller number or shoot dry weight enhanced the root density or root biomass, respectively [4–5]. Interestingly, such root-shoot relationship exists in the vegetative phase but not in the reproductive phase [6–7]. Hence, an extended vegetative phase in winter type growth habit results in more tillers and higher yields in wheat and barley. Contrarily, the spring type growth habit reveals a clear advantage of a shortened vegetative phase and early heading but usually associated with lesser number of yield-producing tillers per plant and shallow root system. It is hypothesized that this relationship may not be simple and there exists a positive feedback loop in the root and shoot development. According to this, an increase of adventitious roots may be associated with a fitness advantage by absorbing more water and nutrients which in turns facilitate more shoot development [8]. The role of a number of genes (like *Vrn-H3*, orthologous to Arabidopsis *FLOWERING LOCUS T*) which facilitate the transition of vegetative to reproductive development have been described in barley. Most of these data are primarily focusing the modulation of spring and winter type growth habits and early heading [9–12]. However, their broader role in plant development (root and shoot development) remain fragmented especially in major agricultural crops like wheat and barley where more tillers and fibrous-deeper rooting are the major determinants of yield, water use efficiency and sustainability.

The wild barley (*Hordeum vulgare* ssp. *sponataneum*) accessions reveal immense variation for root-shoot parameters and water and nutrient absorption which seems to link with their diverse ecological adaptation especially drought stress tolerance [13]. A number of studies have been made to investigate root parameters in wild barley accessions. For instance, Tyagi et al. [14] showed significant differences among 315 wild barley accessions for seminal root number, root length, root fresh weight and root dry weight. Similarly, Grando and Ceccarelli [15] found more vigorous root system in barley landraces and wild accessions than the cultivars. Zhao et al. [16] reported unique variation for root and shoot traits among 47 Tibetian wild barley genotypes. The genetic diversity in wild barley is greater than in cultivars suggesting the potential of wild barley as a source for unique alleles for improvement of agronomic traits as well as for biotic and abiotic stress tolerance [17]. Naz et al. [18,19] utilized a library of introgression lines (S42ILs) originated from an initial cross between spring cultivar Scarlett and wild barley accession ISR42-8 and have identified 15 wild introgressions influencing root and shoot traits under drought stress conditions.

In the present study, we have investigated the genetic inheritance and interplay of root and shoot attributes and their putative association with plant development like heading under the influence of a limited drought period. We have used a second filial (F2) population of a cross between spring barley cultivar Cheri and a unique wild accession ICB181160 adapted to semi-desert conditions of the Middle East. The parental genotypes reveal contrasting root-shoot and heading traits which provide an opportunity i) to dissect the underlying genetic components controlling root, shoot and heading traits ii) to find putative linkages among the selected traits and iii) to better understand the genetic potentials of these traits in agriculture.

## Materials and Methods

### Plant materials

A segregating F2 population derived from a cross between spring barley cultivar Cheri and a wild barley accession ICB181160 was utilized in the present study. Cheri is a German spring barley cultivar (*Hordeum vulgare* ssp. *vulgare*) whereas wild barley accession ICB181160 (*H*. *vulgare* ssp. *spontaneum*) was originating from Iran.

### Experimental setup

The experiment was conducted inside the greenhouse of campus Klein-Altendorf, University of Bonn, Germany. A single seed of each genotype was sown in a plastic pot (19.5 cm diameter × 25.5 cm height) with a capacity of 5.5 L soil containing a mixture of top soil, silica sand, milled lava and peat dust (Terrasoil®, Cordel&Sohn, Salm, Germany). Around three weeks old seedlings were kept outside the greenhouse in the open field for vernalization for six weeks starting from mid-December to end of January under natural cold day and night temperatures. After vernalization, the pots were reshifted to the greenhouse where the day temperature and night temperature were 20°C and 15°C, respectively. The greenhouse was supplied 12 hours (06 till 18 hours) artificial light per day. The water supply was done using table flooding three times (three minutes per irrigation) in a day via a computer mediated irrigation system. The drought stress treatment of F2 population and parental genotypes was carried out at plant development stage BBCH 29–31 by eliminating the water supply completely inside the greenhouse [20]. The development stage BBCH 29–31 stands for end of tillering where maximum number of tillers are detectable to stem elongation. To assess the effect of drought, only the parental genotypes were assigned to control and drought stress blocks. For this, the plants were kept under stress for around 28 days whereas the control block was kept under the continuous supply of irrigation. A list of root and shoot traits were evaluated in the study as presented below:

Root dry weight (RDW): Individual plant roots were dried in an oven for seven days at 50°C and weighed in grams (g).

Root volume (RV): Volume differences before and after immerges of fresh roots in a 500 ml measuring cylinder containing water were measured in cubic centimeters (cm^3^).

Root length (RL): After washing, the fresh root was laid down straight on a table and measured with a ruler in centimeter (cm).

Shoot dry weight (SDW): After harvesting, the above ground individual plant shoots were kept in a perforated plastic bag and dried in an oven for seven days at 50°C. The dry weight was measured in grams (g).

Tiller number per plant (TIL): The total number of tillers were counted for each plant before harvesting.

Days to heading (HEA): The number of days counted until first spike appeared.

### Genotyping and candidate gene analyses

The F2 population containing the parents Cheri and ICB181160 was genotyped using single nucleotide polymorphism (SNP) derived cleaved amplified polymorphic sequence (CAPS) marker system. Polymorphic CAPS markers were established by surveying the parents using SNP sequence data derived from Illumina 9k iSelect Chip from TraitGenetics [9]. By this, a total of 68 CAPS markers distributed across the barley genome were genotyped in F2 population for the initial genetic mapping. The details of these CAPS markers and linkage analysis can be found in Bungartz et al. [21]. Additionally, two gene-specific marker HvVrnH3, HvVrnH1 were developed based on the allelic polymorphism and genotyped in F2 population as well as in the parental genotypes. Similarly, additional candidate genes were genotyped and sequenced using standard PCR and sequencing protocols. The details of primers utilized for genotyping and sequencing of candidate genes are presented in Table A in S1 File.

### Statistical analysis

Trait-wise comparison of means was performed among F2 population, Cheri and ICB181160 using PROCGLM procedure in SAS program version 9.3 [22,23]. For this, the variance components were estimated using PROC VARCOMP procedure. Genetic correlation coefficients (r) between traits among the F2 individuals were estimated using the CORR procedure in SAS. The QTL mapping was carried out in SAS program version 9.3 using PROC GLM by applying the following general linear equation:

Yij=μ+Mi+Lj(Mi)+εij

Where *μ* is the general mean, *M*_*i*_ is the fixed effect of the _*i*_th marker, *Lj*(*Mi*) is the random effect of the _*j*_th F2 line nested in the _*i*_th marker genotype. The single putative QTL was assumed when the significant markers revealed a distance of ≤ 20 centiMorgan (cM) and showed the highest F-value from each group with P value is less than 0.05. The proportion of observed phenotypic variance attributable to a particular QTL was estimated by the coefficient of determination (R^2^) according to Arifuzzaman et al. [24]. The relative performance of the homozygous exotic genotype (RP[*Hsp*]) were calculated using the following formula:

$$\text{RP}[Hsp]=\frac{\left[\text{Lsmeans}(Hsp)-\text{Lsmeans}(Hv)\right]}{\text{Lsmeans}(Hv)}\times 100$$

Interaction plots were made with the R statistics software version 3.1.1 to show the phenotypical distribution of the individuals in the population and effect of the interaction between genes to the phenotype [25]. As *Vrn-H2* showed a null allele, we use a tightly linked marker CAPS124 (111.3 cM) on chromosome 4H to select homozygous and heterozygous alleles among F2 population.

## Results

### Phenotypic characterization

Trait-wise mean comparison among Cheri, ICB181160 and the F2 population is presented in Table 1. Cheri, ICB181160 and the F2 population revealed significant variation for root dry weight (RDW), root volume (RV), root length (RL), shoot dry weight (SDW), tiller number per plant (TIL) and days until heading (HEA) under drought stress conditions.

**Table 1:** Mean comparisons of the root and shoot traits among Cheri, ICB181160 and the F2 population.

Trait^a^	Genotypes	Mean	Minimum	Maximum	Standard error
RDW	Cheri	3.0	1.7	4.5	0.2
	ICB181160	3.7	2.6	5.3	0.2
	F2	4.0	1.1	11.2	0.1
RV	Cheri	28.4	10.0	45.0	3.0
	ICB181160	38.5	25.0	50.0	2.6
	F2	32.5	15.0	100.0	1.0
RL	Cheri	37.6	31.0	43.0	1.0
	ICB181160	40.8	32.0	47.0	1.3
	F2	38.3	21.0	62.0	0.5
SDW	Cheri	138.8	112.2	180.5	7.2
	ICB181160	144.9	103.7	202.9	7.8
	F2	127.4	53.0	193.2	1.9
TIL	Cheri	24.0	17.0	36.0	1.5
	ICB181160	22.0	16.0	34.0	1.5
	F2	22.7	3.0	48.0	0.7
HAE	Cheri	96.1	76.0	105	2.7
	ICB181160	99.7	84.0	129.0	4.0
	F2	105.4	75.0	138.0	1.9

^a^ Trait abbreviations: RDW = root dry weight (g), RV = root volume (cm^3^), RL = root length (cm), SDW = shoot dry weight (g), TIL = number of tillers per plant, HEA = days to heading.

For RDW, the F2 population exhibited higher mean RDW (4.0 g) compared to Cheri (3.0 g) and ICB181160 (3.7 g). Moreover, the F2 population accounted for remarkable variation in RDW that ranged from 1.1 to 11.2 g. The wild accession ICB181160 revealed higher mean RV (38.5 cm^3^) compared to the F2 (32.5 cm^3^) and Cheri (28.4 cm^3^). A wide range of variation (15–100 cm^3^) observed in the F2 population for RV under drought conditions. The highest mean RL (40.8 cm) revealed for ICB181160 whereas the average RL in Cheri and F2 population was 37.6 cm and 38.3 cm, respectively. The F2 population accounted for 62.0 cm maximum RL that was higher than ICB181160 and Cheri. The F2 population showed a remarkable variation for SDW that ranged from 53.0 to 193.2 g giving a mean of 127.4 g. Almost similar mean TIL values were found for all genotypes. The F2 population exhibited higher maximum (48) and lower minimum (3) numbers of TIL. The highest mean HEA was found in the F2 population (105.4) as compared to both parents Cheri and ICB181160.

### Genetic correlations

The correlation coefficients among the traits under drought conditions are presented in Table 2. RDW exhibited highly significant and positive correlations with SDW (0.55) and RL (0.43). RV revealed strong significant correlations with SDW (0.51) and RL (0.56). Among the shoot traits, TIL exhibited a positive and statistically significant correlation with SDW (0.32) and HEA (0.31).

**Table 2:** Correlation coefficients among the root and shoot traits in the F2 population.

Trait^a^	RV	RL	SDW	TIL	HEA
RDW	0.69***	0.43***	0.55***	0.37***	0.36***
RV		0.51***	0.56***	0.33***	0.24**
RL			0.37***	0.20*	ns
SDW				0.32***	0.25**
TIL					0.31***

^a^ Trait abbreviations: RDW = root dry weight (g), RV = root volume (cm^3^), RL = root length (cm), SDW = shoot dry weight (g), TIL = number of tillers per plant, HEA = days to heading.

*, ** and *** indicates level of significance at 5%, 1% and 0.1% levels of probability, respectively. ns = non significant.

### QTL detection

**Fig 1:**
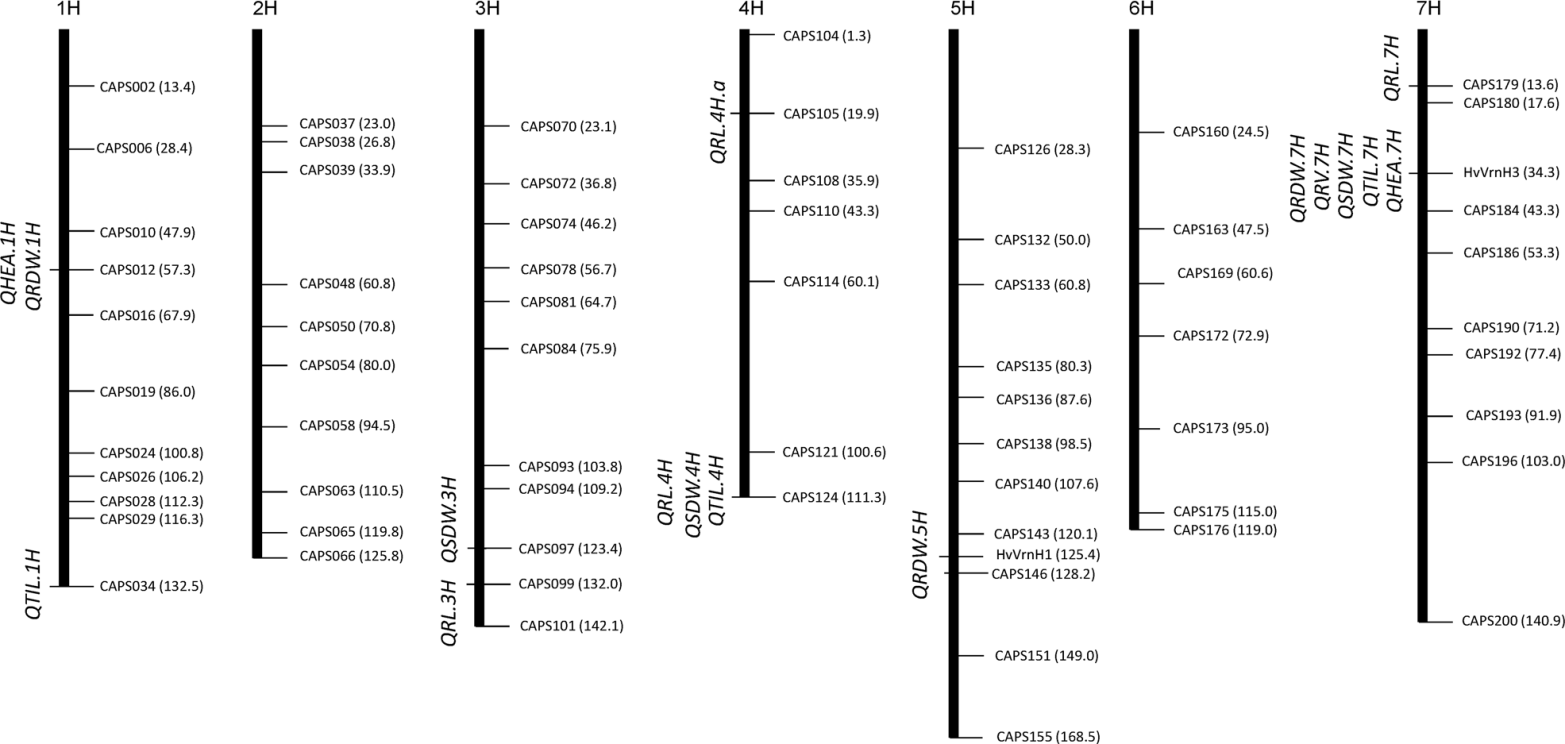
QTL map of root, shoot and heading traits detected in the F2 population of a cross between spring barley cultivar Cheri and wild accession ICB181160.

The QTL analysis revealed 16 putative QTL for shoot and root traits of which twelve and four QTL alleles were linked to the favorable performance of the exotic and elite alleles, respectively (Table 3). These QTL effects were localized on chromosomes 1H, 3H, 4H, 5H and 7H (Fig 1).

**Table 3:** List of detected QTL for the root, shoot and heading traits in the F2 population of a cross between spring barley cultivar Cheri and wild accession ICB181160.

Trait^a^	QTL^b^	Marker^c^	Chr^d^	Position^e^ (cM)	*F-*value^f^	Probability^g^	AA^h^	aa^i^	RP (aa) (%)^j^	R^2^ (%)^k^[44]
RDW	*QRDW*.*1H*	*CAPS165*	1H	57.3	5.6	<0.01	3.4	4.0	19.3	6.5
	*QRDW*.*5H*	*CAPS112*	5H	128.2	3.7	<0.05	3.8	4.6	31.9	3.7
	*QRDW*.*7H*	*HvVrnH3*	7H	34.3	7.7	<0.001	3.4	5.0	48.6	9.0
RV	*QRV*.*7H*	*HvVrnH3*	7H	34.3	12.8	<0.001	28.6	41.3	44.3	13.7
RL	*QRL*.*3H*	*CAPS070*	3H	132	4.3	<0.01	40.5	37.7	-7.1	3.8
	*QRL*.*4H*.*a*	*CAPS076*	4H	19.9	6.2	<0.01	40.1	35.5	-11.3	7.2
	*QRL*.*4H*	*CAPS091*	4H	111.3	4.8	<0.05	37.9	42.3	11.5	4.8
	*QRL*.*7H*	*CAPS144*	7H	13.6	3.1	<0.05	36.4	37.1	1.9	3.9
SDW	*QSDW*.*3H*	*CAPS178*	3H	123.4	3.2	<0.05	121.1	119.4	-1.4	3.8
	*QSDW*.*4H*	*CAPS091*	4H	111.3	4.0	<0.05	124.5	143.8	15.5	4.9
	*QSDW*.*7H*	*HvVrnH3*	7H	34.3	4.4	<0.01	119.6	136.3	13.9	5.2
TIL	*QTIL*.*1H*	*CAPS023*	1H	132.5	3.2	<0.05	24.9	24.7	-0.8	3.8
	*QTIl*.*4H*	*CAPS091*	4H	111.3	3.2	<0.05	19.8	24.5	23.8	3.8
	*QTIL*.*7H*	*HvVrnH3*	7H	34.3	10.3	<0.001	17.7	26.4	49.2	10.7
HEA	*QHEA*.*1H*	*CAPS165*	1H	57.3	7.5	<0.001	101.3	106.0	4.6	8.0
	*QHEA*.*7H*	*HvVrnH3*	7H	34.3	11.7	<0.001	100.4	110.1	9.7	12.6

^a^ Trait abbreviations: RDW = root dry weight (g), RV = root volume (cm^3^), RL = root length (cm), SDW = shoot dry weight (g), TIL = number of tillers per plant, HEA = days to heading.

^b^ Description of quantitative trait locus.

^c^ Linked CAPS marker.

^d^ Chromosome.

^e^ Marker position in CentiMorgan.

^f^ *F* value of the given marker locus.

^g^ Probability at <0.05, <0.01 and <0.001 levels.

^h^ Least square means of homozygous allele in Cheri.

^i^ Least square means of homozygous allele in ICB181160.

^j^ Relative trait performance of the exotic allele compared to cultivar Cheri, calculated as RP(aa) % = [Lsmeans(aa)–Lsmeans(AA)]/ Lsmeans(AA) *100.

^k^ Genetic variance explained by marker.

Three putative QTL for RDW were mapped on chromosomes 1H, 5H and 7H. The exotic QTL alleles at *QRDW*.*1H*, *QRDW*.*5H* and *QRDW*.*7H* revealed an increase in RDW ranged from 19.3% to 48.6%. Among these, the strongest QTL *QRDW*.*7H* was detected on chromosome 7H at the putative region of *VrnH3* gene which accounted for 48.6% increase in RDW and explained 9.0% of the genetic variance. This QTL region also showed the strongest performance for RV, SDW, TIL and HEA. Four putative QTL were detected for RL and located on chromosomes 3H, 4H and 7H. According to the relative performance of the exotic allele, two QTL at *QRL*.*3H* and *QRL*.*4H*.*a* resulted in the reduction of RL ranging from 7.1 and 11.3%. The exotic QTL alleles at *QRL*.*4H* and *QRL*.*7H* increased RL by 11.5% and 1.9%, respectively. For SDW, three putative QTL were located on chromosomes 3H, 4H and 7H. The strongest QTL effect (*QSDW*.*7H*) was linked to the marker locus *VrnH3* which accounted for 13.9% increase in SDW and explained 5.2% of the genetic variance. At *QSDW*.*3H*, the performance of the elite alleles was higher than the exotic alleles by 1.4% and accounted 3.8% genetic variance. Three QTL for TIL were detected and located on chromosomes 1H, 4H and 7H. The strongest QTL for TIL was linked to the gene-specific marker locus *VrnH3* that increased TIL due to the exotic allele performance by 49.2%. Two major QTL for HEA were located on chromosomes 1H and 7H. The strongest QTL *QHEA*.*7H* were linked to marker HvVrnH3 in positions 34.3cM on 7H chromosome. The exotic allele (aa) revealed about 10 days late heading than elite allele (AA) and explained 12.6% genetic variance.

### Candidate gene analysis

**Fig 2:**
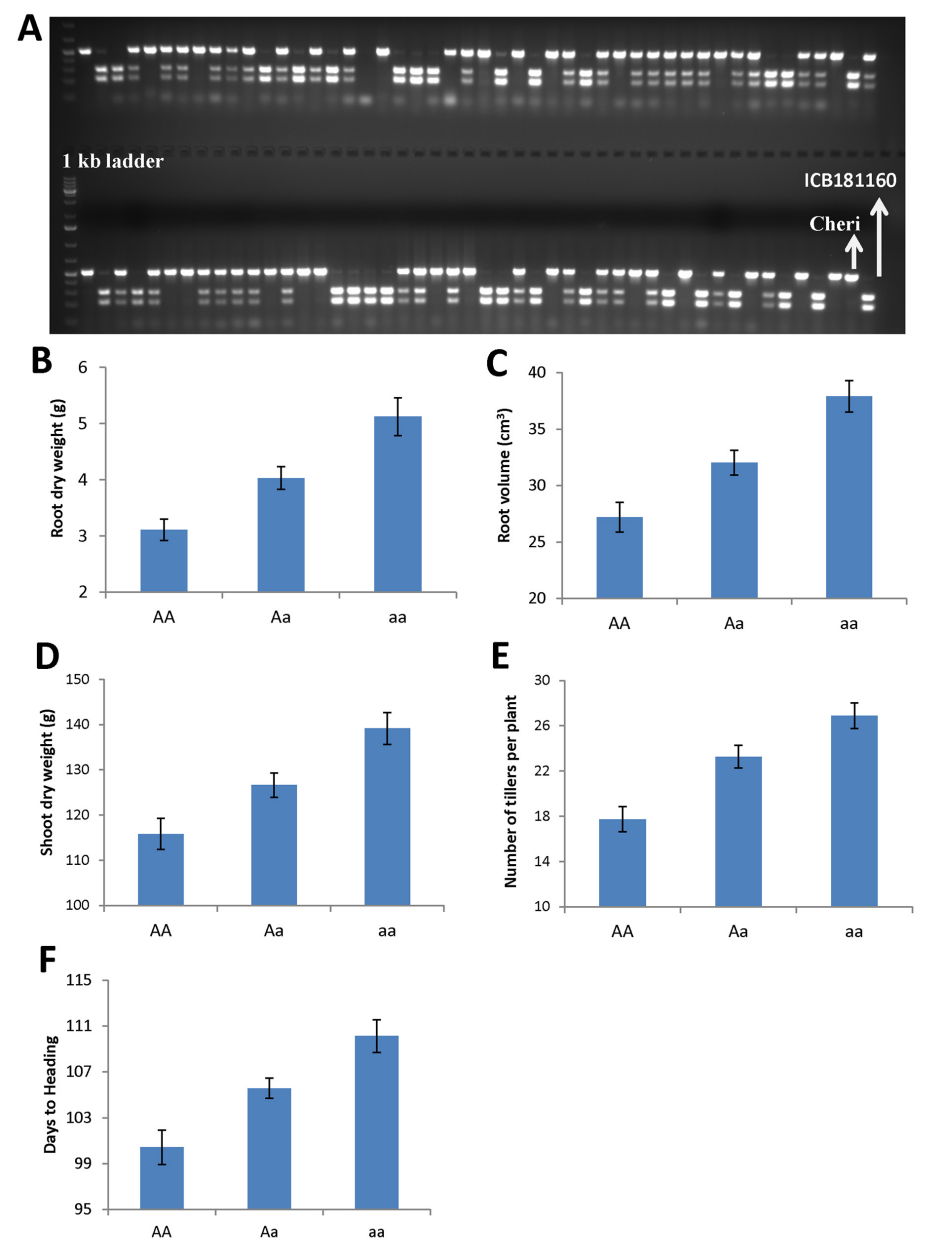
Genotypic and phenotypic trait variations in the F2 population for the gene-specific marker HvVrnH3. A) Genotypic scores of marker HvVrnH3 showing polymorphic *Vrn-H3* and *vrn-H3* alleles in barley. B-F) Phenotypic quantification of QTL showing the common effect of *Vrn-H3* gene on root, shoot and heading traits. Cultivar Cheri allele (AA), heterozygous (Aa) and ICB181160 allele (aa).

**Fig 3:**
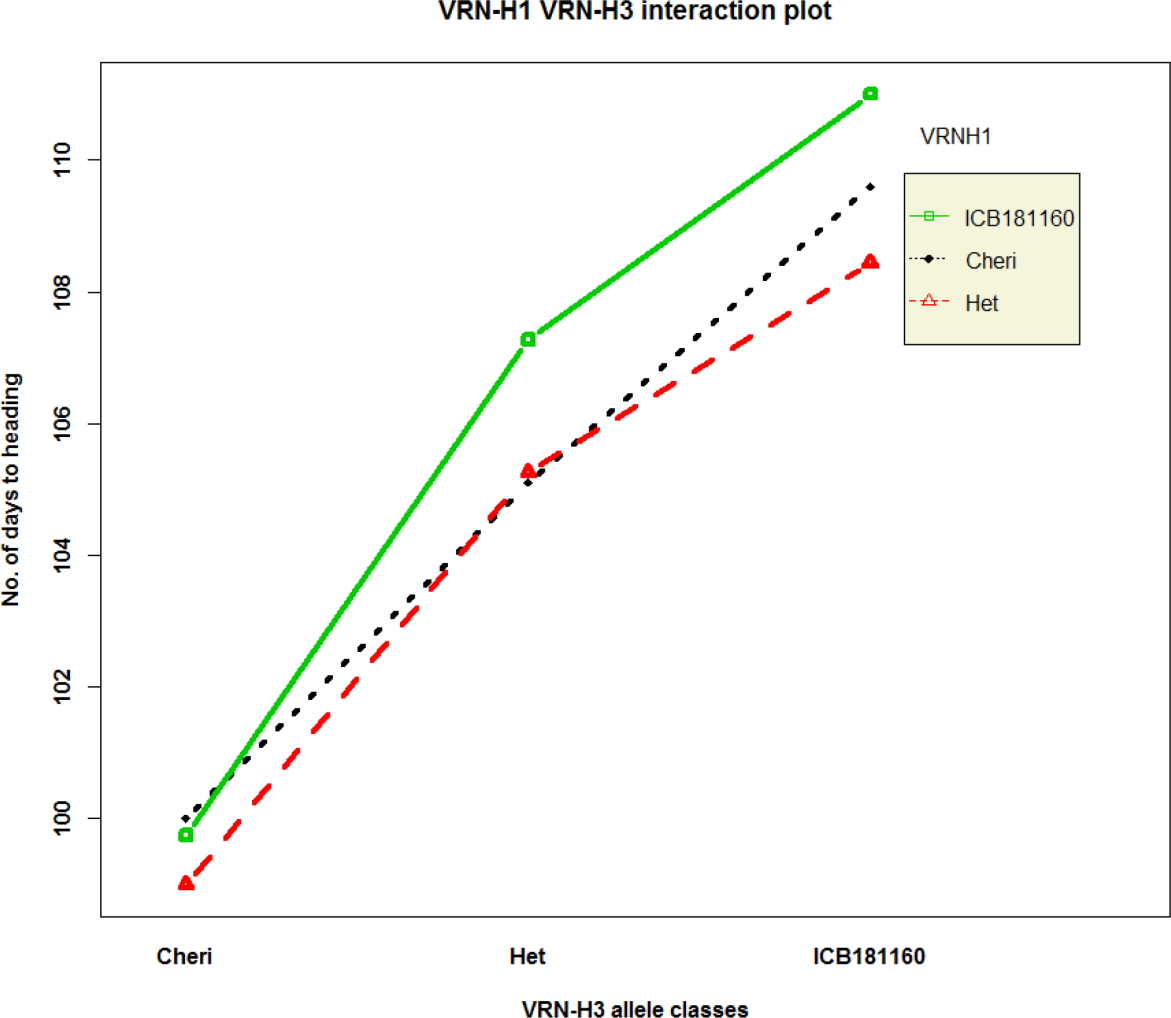
Genetic interaction plot of *Vrn-H3* and *Vrn-H1* alleles from Cheri and ICB181160 among the F2 population for heading. All nine allele combinations of both genes are calculated using R-interaction plots.

**Fig 4:**
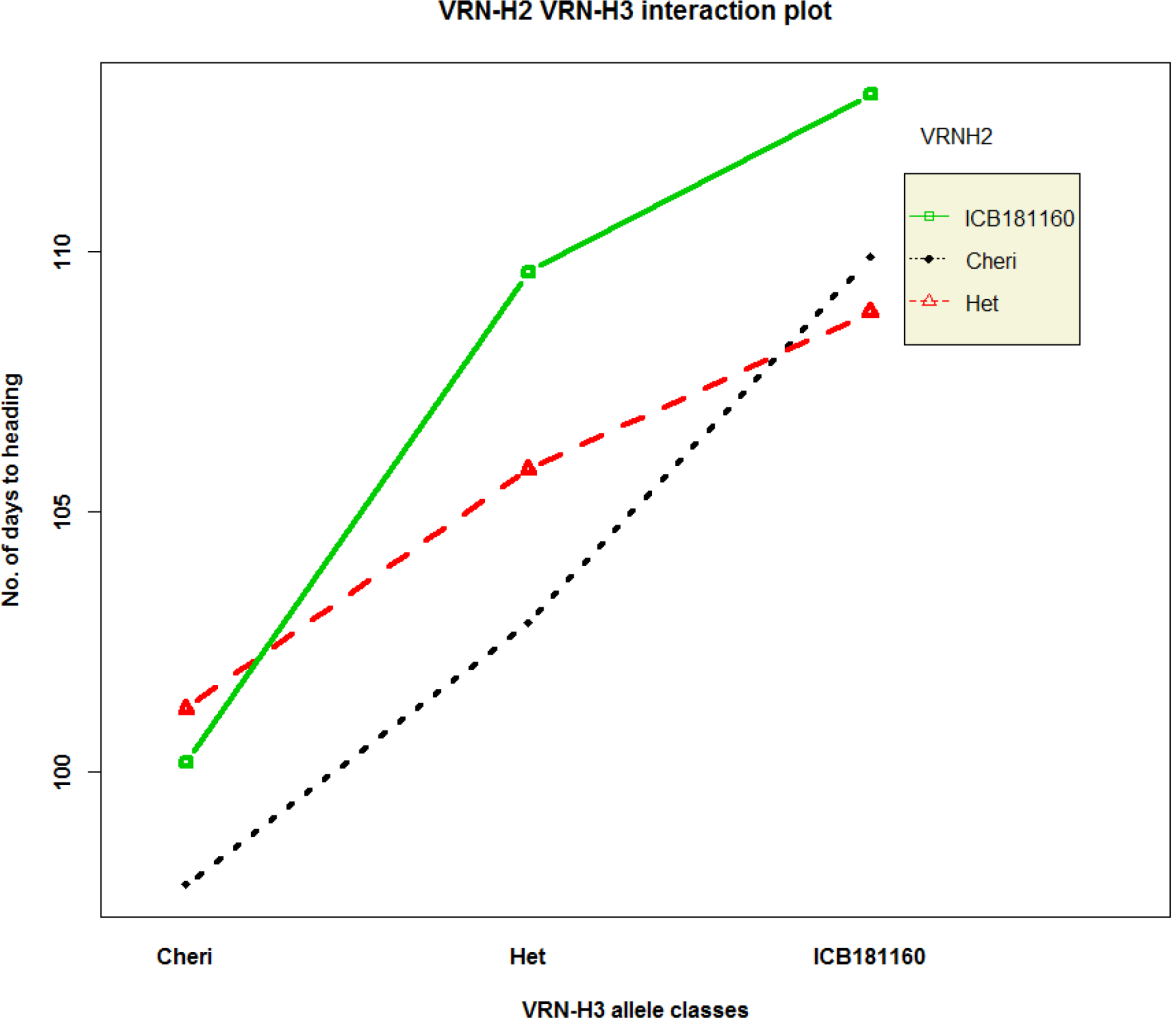
Genetic interaction plot of *Vrn-H3* and *Vrn-H2* alleles from Cheri and ICB181160 among F2 population for heading. All nine allele combinations of both genes are calculated using R-interaction plots.

QTL analysis reveals a common and major QTL on 7HS chromosome for SDW, RDW and RV and HAE at the putative region of *Vrn-H3* gene. To confirm if this common QTL effect was due to *Vrn-H3* gene, we developed a gene specific CAPS marker (HvVrn-H3) from the previously described SNPs that showed the polymorphism between the early heading allele *Vrn-H3* and late allele *vrn-H3* and genotyped the F2 population and parental genotypes. This analysis revealed a clear segregation of early heading *Vrn-H3* allele (Cheri, AA) and late heading *vrn-H3* allele (ICB181160, aa) in the F2 population and parental genotypes (Fig 2A). Notably, the early and late heading phenotypes were correlated with lower and higher root-shoot attributes, respectively (Fig 2B–2F). Furthermore, we sequenced the full-length gene to find putative mutations between Cheri and ICB181160. Sequence analysis of both parental genotypes and their alignment with already described haplotypes among the reference genotypes indicated that the intron 1 haplotype A-G was associated with late flowering and haplotype T-C with early flowering, thus confirmed already described mutation in intron 1 in the present study (Table 4, Casas et al. [26]). In addition, we found two novel SNPs in position 80 and 302 and unique 4 bp deletion at position 326 in intron 1 in ICB181160. Based on the existing description by Casas et al. [26] and polymorphisms detected in the present study, we designated early heading Cheri allele as *Vrn-H3* and late flowering ICB181160 allele as *vrn-*H3 (Table 4 and Figure A in S1 File). To elucidate the genetics behind early and late heading in Cheri and ICB181160, we also genotyped gene-specific marker HvVrnH1 in F2 population, which amplifies a 400 bp fragment in Cheri and 580 bp fragment in ICB181160 which was located in intron 1 of the *VRN-H1* gene. This marker polymorphism indicated that Cheri has the dominant *Vrn-H1* allele (early flowering) whereas ICB181160 bears the recessive *vrn-H1* allele (late flowering). Further, genotyping and sequencing of the full-length *Vrn-H2* gene in parental genotypes showed that Cheri bore a null allele *vrn-h2* (early flowering). Contrarily, wild barley accession harbored dominant *Vrn-H2* allele (late flowering) (Tables B and C in S1 File). These data thus suggest a predominant role of vernalization pathway in the determination of major QTL effect in the present study. Therefore, we calculated the genetic interactions among the members of vernalization pathway genes, *Vrn-H3*, *Vrn-H1* and *Vrn-H2* in the F2 population. For this, the effect of pairwise allelic combinations on heading and root/shoot trait were calculated using R-interaction plots. This results showed that homozygous *vrn-H3* alleles of ICB181160 resulted in delayed heading in the background of homozygous (*Vrn-H1*/*Vrn-H1*) or heterozygous (*Vrn-H1/vrn-H1*) alleles among the F2 plants (Fig 3). Similarly, we found interaction between *Vrn-H3* and *Vrn-H2* alleles where homozygous *vrn-H3*/*vrn-H3*:*Vrn-H2/Vrn-H2* alleles combination from ICB181160 were associated with delayed heading. Whereas, the F2 plants having homozygous *Vrn-H3/Vrn-H3*:*vrn-H2/vrn-H3* alleles combination from Cheri showed highly early heading phenotypes (Fig 4). The allelic combinations associated with delayed heading were also correlated with higher RDW and RV and RL and TIL values. Whereas, the allelic combinations associated with early heading were showing lower trait values of root/shoot traits (Figures B, C, D, E and F in S1 File).

**Table 4:** Barley haplotype scoring from six SNPs and one indel in the intron 1 and exon 2 of *Vrn-H3* gene.

**Cultivar/accession**	**Position in intron 1**						**in Exon 2**	** **	**Accession Number**
	**63**	**80**	**270**	**302**	326^a^	**384**	**36**	Allele^b^	
Triumph	T	C	**T**	A	in	C	C	*Vrn*-*H3*	* DQ898520*
Strider	C	C	**A**	A	in	**G**	C	*vrn*-*H3*	* EU007830*
Cheri	C	C	**T**	A	in	**C**	C	*Vrn-H3*	* This study*
ICB181160	C	T	**A**	T	del	G	T	*vrn*-*H3*	* This study*

^a^ “in” and “del” refers a 4 bp indel GCTC.

^**b**^ Alleles linked with early and late heading respectively entitled as *“Vrn-H3”* from Cheri and *“vrn-H3”* from ICB181160.

We also sequenced the photoperiod genes *PPDH2*, *HvELF3*, *HvCCA1*, *HvLUX1* in parents which showed no allelic differences for early and late heading alleles between Cheri and ICB181160 (Tables D, E and Text A in S1 File). However, *PPDH1* and *HvCEN* revealed early and late flowering alleles polymorphisms in Cheri and ICB181160, respectively (Table D and Text A).

## Discussion

The wild barley accessions bear an inherent ability to develop a fibrous and deep rooting which is usually linked with shoot biomass. These features of wild barley seem to link with its natural fitness and adaptation across diverse environmental conditions [15,14]. The fibrous root system is advantageous in term of water and nutrients use efficiency, for instance reaching immobile soil resources (like phosphorus) as well as it is associated with crucial shoot development traits. On the other hand, the magnitude of these adaptive traits were low in the cultivated varieties which show an inferior and less fibrous shallow root system putatively lost during domestication. This lacking seriously undermines the water and nutrient use efficiency as well as sustainability of the modern varieties. The present study was aimed to identify valuable alleles of a unique wild barley accession revealing extensive fibrous deeper root system. Along with the root traits, we have measured important shoot and heading traits primarily to dissect putative linkage of these traits to better understand the importance of these traits for crop production.

The F2 population revealed significant variation for RDW, RV, RL, SDW, TIL and HEA indicating an appropriate segregation of wild segments in this population. Additionally, we got a number of phenotypic classes for all traits among the F2 population suggesting quantitative inheritance of root and shoot attributes. Moreover, the F2 plants revealed transgressional effect for most of the root and shoot traits. Wu et al. [27] found transgressive segregation for RDW in rice RIL population. Plant above ground biomass is primarily a product of photosynthesis, water and mineral nutrients as basic blocks. In barley, the above ground biomass is the product of shoot apical meristem consists of tiller, leaves, stem and inflorescences. Under conditions of drought, the wild accession ICB181160 revealed greater SDW, more TIL and delays of HEA compared to cultivar Cheri (Table 1). F2 population revealed the wide range of variation of these traits. In advanced backcross population, Arifuzzaman et al. [24] revealed higher mean SDW in wild type ISR42-8 compared to cultivar Scarlett. The wild barley acts normally like winter type depending on growth habit in which heading is delayed as compared to spring type growth habit [28]. The delays in heading increases the vegetative phase and plant produced more secondary tillers and shoot biomass. In relation with root parameters more tillers, increased the number of nodal roots [29]. Taken together, plants with a greater shoot biomass show more fibrous root system which enabled them to maintain more water acquisition, gas exchange and carbon assimilation levels and mineral nutrient uptake during water stress conditions [30–32].

The correlation analysis indicated strong positive relationships among the root and shoot traits. The root traits, RDW and RV tend to correlate well with each other [33]. The RDW revealed strongest correlation with RV (0.69). These results suggested that the likelihood of common genetic components influencing these traits simultaneously. RDW revealed strong-positive correlations with RV and SDW. Abdel-Ghani et al. [5] found stronger correlations (0.78) between RDW and SDW in barley. Narayanan et al. [34] also reported similar results in wheat. RV revealed stronger correlations with SDW. Plants with more root volume had higher nutrient concentrations, suggesting that increased root biomass per unit of soil area resulting increased shoot weight under drought conditions [35,36]. It has been reported that the total number of nodes per plant determines the total number of roots [37]. In our study, TIL revealed significant positive correlations with SDW, RDW and RV.

**Fig 5:**
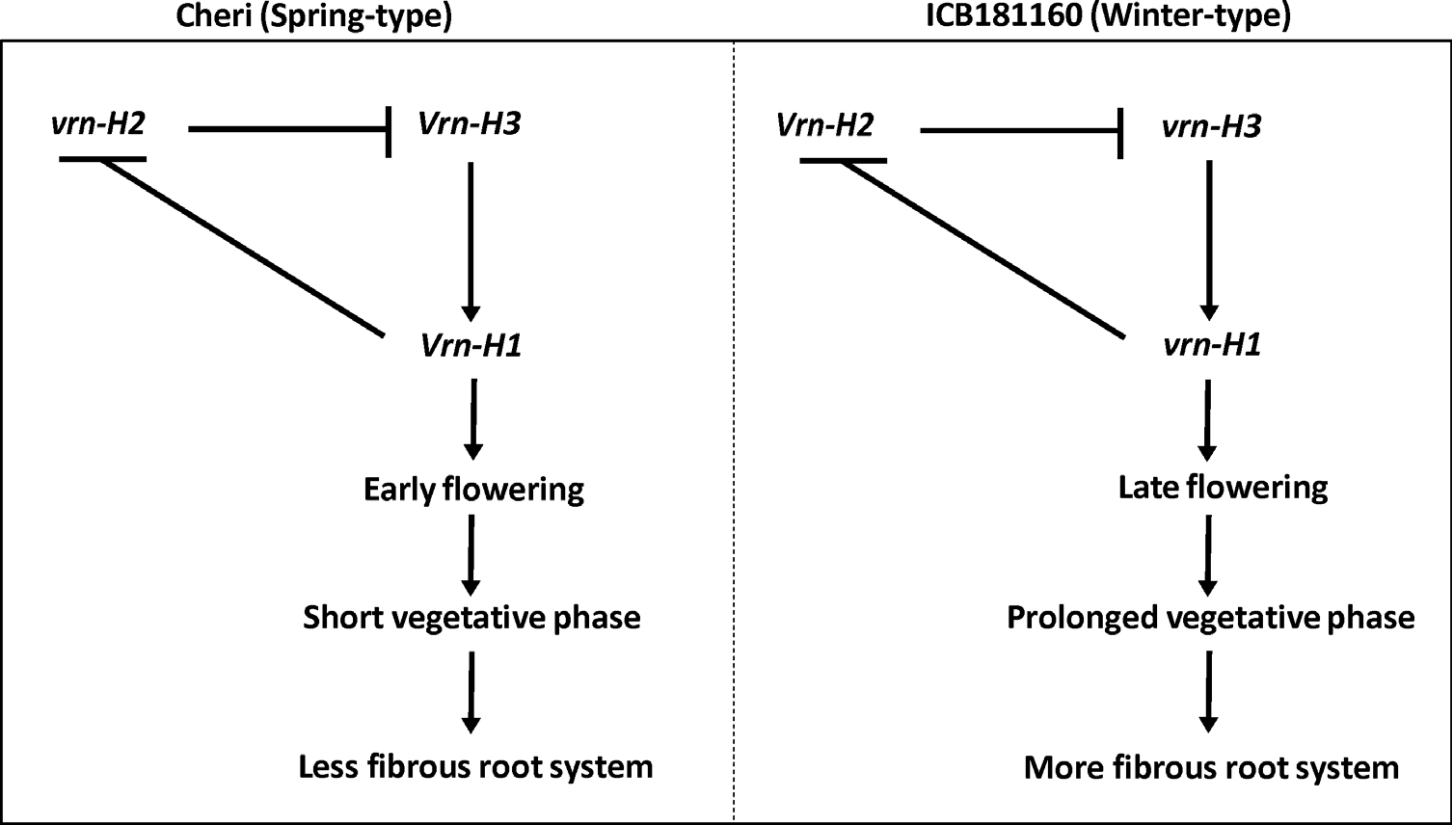
Proposed genetic model suggesting and interplay of heading and root-shoot traits in barley.

Marker by trait analysis revealed altogether eight chromosomal regions associated with root and shoot traits (Fig 1). The strongest QTL was detected on chromosome 7H at the putative region of *Vrn-H3* gene. This QTL showed the effect on RDW, RV, SDW, TIL and HEA simultaneously. This fact lead us to believe the role of major gene *Vrn-H3* in the process of root and shoot development of barley. The *Vrn-H3* is a vernalization gene which is orthologous to Arabidopsis *FLOWERING LOCUS T* (*FT*) and wheat *Vrn-B3* gene [12]. Yan et al. [12] described the causative mutations that differentiate between the early and late flowering alleles of *Vrn-H3* in intron 1. However, later studies on the functional characterization *Vrn-H3* alleles found more complex polymorphisms based on expressional divergence and gene copy number as well as environment dependent genetic regulation [38,39,40]. These data showed that there exist a considerable confusion about the classification of *Vrn-H3* alleles as well as about the causative mutation for the allelic variants among the natural populations. Therefore, to test the putative role of *Vrn-H3* we genotyped a diagnostic PCR based CAPS HvVrn-H3 among the F2 population. This marker show polymorphism and clear co-segregation of early heading *Vrn-H3* allele in Cheri and late heading allele *vrn-H3* in ICB181160 with early and late heading phenotypes. Interestingly, the early and late heading alleles were correlated with the inferior root system (less fibrous) and more fibrous root systems, respectively (Fig 2). It is noteworthy to mention that full-length sequence analysis of *Vrn-H3* confirmed already described mutation from Casas et al. [26] as well as found additional 4 bp deletion in intron 1 of ICB181160. Wang et al. [41] also found this 4 bp deletion in another wild barley accession ISR42-8. Hence, we suggest that this 4 bp deletion in winter-type genotypes may be a better diagnostic marker in deciding early and late heading alleles of *Vrn-H3* among natural populations. Furthermore, the putative role of this deletion needs to be investigated in determining early and late heading in barley. We also tested the interaction of *Vrn-H3*, *Vrn-H1* and *Vrn-H2* using F2 population. This analysis showed that *Vrn-H3* was epistatic to *Vrn-H1* which confirmed its association with major QTL on chromosome 7H as well as in line with existing relationship of vernalization gene in modulating heading in cereals. *Vrn-H3* appeared as the central regulator that drive *Vrn-H1* to initiate flowering [12,39,40]. However, there exists a repression of *Vrn-H2* on *Vrn-H3* in winter-type barley genotypes. This repression is normally released through cold temperature (vernalization) which resulted in the up-regulation of *Vrn-H3* that activates *Vrn-H1* to initiate flowering. The spring-type cultivars where the *vrn-H2* is mutated do not need vernalization for expression of *Vrn-H3* and *Vrn-H1*. The wild barley accession ICB181160 contained recessive *vrn-H1* allele due to huge insertion (>5 kb) in intron 1, which seems to have a regulatory role in the prolonged vegetative growth and delayed flowering indicating two fundamental barriers in early flowering in ICB181160. However, in spring barley genotypes Cheri, there exists a null *vrn-H2* allele having no suppression on *Vrn-H3* that results in short vegetative phase and early flowering through the positive regulation of *Vrn-H3*/*Vrn-H1*. These data help us to propose a putative link of heading and root-shoot traits by which the prolonged and short vegetative phases were linked with the fibrous and inferior root systems in ICB181160 and Cheri, respectively (Fig 5). These findings hence uncover the influence of *Vrn-H3* gene beyond heading, in the process of root and shoot development. These result may also open a new debate or question about the usefulness of early heading in crop production. The early heading genotypes in wheat and barley supposed to produce lesser biomass in term of lesser yield producing tillers which in turn account for shallow rooting system for water use efficiency and sustainability in these crops. However, further experiments are needed to precisely investigate the effect of individual traits on yield under control and drought conditions.

Sequencing of two genes *PPDH1* and *HvCEN* associated to photoperiod also showed polymorphims in the parental genotypes. Both genes were localized to chromosome 2H where no QTL effect was detected in the present study. We believe a primary reason behind this may lie in the short day growing conditions in the present study as both genes showed their major phenotypic effects under long day conditions. Two more QTL regions were found for higher root attributes which were localized on chromosome 1H and 5H. The QTL on chromosome 1H also showed association with heading which seems to reveal a direct relationship of root and heading. In similar positions, Naz et al. [19] and Hoffmann et al. [42] identified QTL for RDW due to the preeminence of exotic allele in the cultivar barley Scarlett background.

The QTL on chromosome 5H revealed a unique effect of an exotic allele by increasing the RDW by 31.9%. This finding is line with Arifuzzaman et al. [24] where they identified RDW QTL due to the exotic allele performance in 301 backcross DH population. Considering the QTL position, it is revealed that the candidate vernalization gene *Vrn-H1* lies on the long arm of chromosome 5H [11,43] in barley. In this region, there was a 5.2 kb deletion on intron 1 in the dominant *Vrn-H1* allele of relative to the wild recessive *vrn-H1* allele. Therefore, we suggest the recessive allele of *vrn-H1* leads to prolonged vegetative phase and more root biomass in the F2 population.

Taken together, the present study has successfully identified QTL for root, shoot and heading traits as well as uncover a primary insight on the interplay of root, shoot and heading in barley. These data presents a first reported on the role of *Vrn-H3* beyond heading, on the essential agronomic traits. We believe these findings will lay the basis for better understanding of barley plant development as well as the planing of future research to investigate a precise role of these attributes in crop production.

## Supporting Information

S1 File Figure A, The alignment of *Vrn-H3* genes between Strider, Triumph, Cheri and ICB181160 with MAFFT version 7. SNPs are with grey background. Figure B, Genetic interaction plot of *Vrn-H2* and *Vrn-H3* alleles from Cheri and ICB181160 for RDW (root dry weight). All nine possible allele combinations of both genes are plotted using R-interaction plot. Figure C, Genetic interaction plot of *Vrn-H1* and *Vrn-H3* alleles for RV (root volume). All nine possible allele combinations of both genes are plotted using R-interaction plot. Figure D, Genetic interaction plot of *Vrn-H2* and *Vrn-H3* alleles for RV (root volume). All nine possible allele combinations of both genes are plotted using R-interaction plot. Figure E, Genetic interaction plot of *Vrn-H2* and *Vrn-H3* alleles for RL (root length). All nine possible allele combinations of both genes are plotted using R-interaction plot. Figure F, Genetic interaction plot of *Vrn-H2* and *Vrn-H3* alleles for TIL (no. of tillers). All nine possible allele combinations of both genes are plotted using R-interaction plot. Table A, List of primers used for the genotyping and sequencing of the candidate genes. Forward (F) and reverse orientation (R) primers. Table B, Barley haplotype scoring from five non-synonymous SNPs and one SSR (simple sequence repeat) in the exon1 and exon 2 of *ZCCT-Ha* gene. Table C, Barley haplotype scoring from six non synonymous SNPs in the exon1 and exon 2 of *ZCCT-Hb* gene. Table D, Barley haplotype scoring from two described SNPs in the exon8 of *PPDH1* gene. Table E, Barley haplotype scoring from two SNPs in the intron 3 of *PPDH2* gene. Table F, Barley haplotype scoring from nine SNPs and three indels in the exon 2 and intron 2 of *HvELF3* gene. Text A, Description of *HvCCA1*, *HvLUX1* and *HvCEN* alleles in cultivar Cheri and ICB181160.
(PDF)

## References

[1] Easlon HM, Bloom AJ. The effects of rising atmospheric carbon dioxide on shoot-root nitrogen and water signaling. Frontiers in Plant Science. 2013;4 10.3389/fpls.2013.00304 PMC3739423 23983674

[2] Hackett C. A study of the root system of barley. I. Effects of nutrition on two varieties. New Phytologist. 1968;67: 287–299. 10.1111/j.1469-8137.1968.tb06384.x

[3] Fageria NK, Moreira A. The role of mineral nutrition on root growth of crop plants. Advances in Agronomy. 2011;: 251–331. 10.1016/b978-0-12-385531-2.00004-9

[4] Hockett EA. Relationship of adventitious roots and agronomic characteristics in barley. Canadian Journal of Plant Science. 1986;66: 257–280. 10.4141/cjps86-040

[5] Abdel-Ghani AH, Neumann K, Wabila C, Sharma R, Dhanagond S, Owais SJ, et al Diversity of germination and seedling traits in a spring barley (*Hordeum vulgare* L.) collection under drought simulated conditions. Genetic Resources and Crop Evolution. 2014;62: 275–292. 10.1007/s10722-014-0152-z

[6] Kwiatkowska D. Flowering and apical meristem growth dynamics. Journal of Experimental Botany. 2008;59: 187–201. 10.1093/jxb/erm290 18256052

[7] Watt M, Moosavi S, Cunningham SC, Kirkegaard JA, Rebetzke GJ, Richards RA. A rapid, controlled-environment seedling root screen for wheat correlates well with rooting depths at vegetative, but not reproductive, stages at two field sites. Annals of Botany. 2013;112: 447–455. 10.1093/aob/mct122 23821620 PMC3698392

[8] Gahoonia TS, Nielsen NE. Root traits as tools for creating phosphorus efficient crop varieties. Plant and Soil. 2004;260: 47–57. 10.1023/b:plso.0000030168.53340.bc

[9] Comadran J, Kilian B, Russell J, Ramsay L, Stein N, Ganal M, et al Natural variation in a homolog of *Antirrhinum CENTRORADIALIS* contributed to spring growth habit and environmental adaptation in cultivated barley. Nature Genetics. 2012;44: 1388–1392. 10.1038/ng.2447 23160098

[10] Fu D, Szűcs P, Yan L, Helguera M, Skinner JS, Zitzewitz JV, et al Large deletions within the first intron in VRN-1 are associated with spring growth habit in barley and wheat. Molecular Genetics and Genomics. 2005;273: 54–65. 10.1007/s00438-004-1095-4 15690172

[11] Mohammadi M, Torkamaneh D, Nikkhah H-R. Correlation of vernalization loci *VRN-H1* and *VRN-H2* and growth habit in barley germplasm. International Journal of Plant Genomics. 2013;2013: 1–9. 10.1155/2013/924043 PMC3628217 23606828

[12] Yan L, Fu D, Li C, Blechl A, Tranquilli G, Bonafede M, et al The wheat and barley vernalization gene *VRN3* is an orthologue of *FT*. Proceedings of the National Academy of Sciences. 2006;103: 19581–19586. 10.1073/pnas.0607142103 PMC1748268 17158798

[13] Nevo E, Chen G. Drought and salt tolerances in wild relatives for wheat and barley improvement. Plant, Cell & Environment. 2010;33: 670–685. 10.1111/j.1365-3040.2009.02107.x 20040064

[14] Tyagi K, Lee HJ, Lee CA, Steffenson BJ, Kim YJ, Yun SJ. Variation in seedling root traits in wild barley (*Hordeum vulgare* L. *ssp*. *spontaneum*) germplasm. Plant Genetic Resources. 2014;12 10.1017/s1479262114000641

[15] Grando S, Ceccarelli S. Seminal root morphology and coleoptile length in wild (*Hordeum vulgare ssp*. *spontaneum*) and cultivated (*Hordeum vulgare ssp*. *vulgare*) barley. Euphytica. 1995;86: 73–80.

[16] Zhao J, Sun H, Dai H, Zhang G, Wu F. Difference in response to drought stress among Tibet wild barley genotypes. Euphytica. 2009;172: 395–403. 10.1007/s10681-009-0064-8

[17] Budak H, Kantar M, Kurtoglu KY. Drought tolerance in modern and wild wheat. The Scientific World Journal. 2013;2013: 1–16. 10.1155/2013/548246 PMC3671283 23766697

[18] Naz AA, Ehl A, Pillen K, Léon J. Validation for root-related quantitative trait locus effects of wild origin in the cultivated background of barley (*Hordeum vulgare* L.). Plant Breeding. 2012;131: 392–398. 10.1111/j.1439-0523.2012.01972.x

[19] Naz AA, Arifuzzaman M, Muzammil S, Pillen K, Léon J. Wild barley introgression lines revealed novel QTL alleles for root and related shoot traits in the cultivated barley (*Hordeum vulgare* L.). BMC Genetics. 2014;15 10.1186/s12863-014-0107-6 PMC4200126 25286820

[20] Lancashire PD, Bleiholder H, Boom TVD, Langelüddeke P, Stauss R, Weber E, et al A uniform decimal code for growth stages of crops and weeds. Annals of Applied Biology. 1991;119: 561–601. 10.1111/j.1744-7348.1991.tb04895.x

[21] Bungartz A, Klaus M, Mathew B, Léon J, Naz AA. Development of new SNP derived cleaved amplified polymorphic sequence marker set and its successful utilization in the genetic analysis of seed color variation in barley. Genomics. 2016;107: 100–107. 10.1016/j.ygeno.2015.12.007 26738469

[22] SAS Institute. The SAS Enterprise Guide for Windows, 9.3 Cary, NC, USA SAS Institute Inc; 2011.

[23] Holland JB, Nyquist WE, Cervantes-Martínez CT. Estimating and Interpreting Heritability for Plant Breeding: An Update. Plant Breeding Reviews. 2003;22: 9–112. 10.1002/9780470650202.ch2

[24] Arifuzzaman M, Sayed MA, Muzammil S, Pillen K, Schumann H, Naz AA, et al Detection and validation of novel QTL for shoot and root traits in barley (*Hordeum vulgare* L.). Molecular Breeding. 2014;34: 1373–1387. 10.1007/s11032-014-0122-3

[25] R Core Team. R: A language and environment for statistical computing. R Foundation for Statistical Computing, Vienna, Austria; 2013.

[26] Casas AM, Djemel A, Ciudad FJ, Yahiaoui S, Ponce LJ, Contreras-Moreira B, et al *HvFT1* (*VrnH3*) drives latitudinal adaptation in Spanish barleys. Theoretical and Applied Genetics. 2011;122: 1293–1304. 10.1007/s00122-011-1531-x 21279626

[27] Wu QS, Wan XY, Su N, Cheng ZJ, Wang JK, Lei CL, et al Genetic dissection of silicon uptake ability in rice (*Oryza sativa* L.). Plant Science. 2006;171: 441–448. 10.1016/j.plantsci.2006.05.001 25193641

[28] Nishida H, Ishihara D, Ishii M, Kaneko T, Kawahigashi H, Akashi Y, et al Phytochrome C is a key factor controlling long-day flowering in barley. Plant Physiology. 2013;163: 804–814. 10.1104/pp.113.222570 24014575 PMC3793059

[29] Kuhlmann H, Barraclough PB. Comparison between the seminal and nodal root systems of winter wheat in their activity for N and K uptake. Zeitschrift für Pflanzenernährung und Bodenkunde Z Pflanzenernaehr Bodenk. 1987;150: 24–30. 10.1002/jpln.19871500106

[30] Kage H, Kochler M, Stützel H. Root growth and dry matter partitioning of cauliflower under drought stress conditions: measurement and simulation. European Journal of Agronomy. 2004;20: 379–394. 10.1016/s1161-0301(03)00061-3

[31] White PJ, Bengough AG, Bingham IJ, George TS, Karley AJ, Valentine TA. Induced mutations affecting root architecture and mineral acquisition in barley. Induced plant mutations in the genomics era Rome: Food and Agricultural Organization of the United Nations 2009; 338–340.

[32] Lynch JP. Steep, cheap and deep: an ideotype to optimize water and N acquisition by maize root systems. Annals of Botany. 2013;112: 347–357. 10.1093/aob/mcs293 23328767 PMC3698384

[33] Haase DL. 2011. Seedling root targets. In: Riley LE, Haase DL, Pinto JR, technical coordinators. National Proceedings: Forest and Conservation Nursery Associations—2010; Proc. RMRS-P-65.

[34] Narayanan S, Mohan A, Gill KS, Prasad PVV. Variability of root traits in spring wheat germplasm. PLoS ONE. 2014;9 10.1371/journal.pone.0100317 PMC4063797 24945438

[35] Haase DL, Rose R. Effects of soil water content and initial root volume on the nutrient status of 2+0 Douglas-fir seedlings. New Forests. 1994;8: 265–277. 10.1007/bf00025372

[36] Sundström E, Keane M. Root architecture, early development and basal sweep in containerized and bare-rooted Douglas fir (*Pseudotsuga menziesii*). The Supporting Roots of Trees and Woody Plants: Form, Function and Physiology. 2000;: 83–96. 10.1007/978-94-017-3469-1_8

[37] Shin JC, Lee MH, Kwon YW. Root-shoot relationship in rice. In Proceedings the 3rd JSSR Symposium on Ideotype of Root System in Rice. Fukui, Japan: World Scientific Pub Co Inc.1995.

[38] Hemming MN, Peacock WJ, Dennis ES, Trevaskis B. Low-temperature and daylength cues are integrated to regulate *FLOWERING LOCUS T* in barley. Plant Physiology. 2008;147: 355–366. 10.1104/pp.108.116418 PMC2330320 18359843

[39] Loscos J, Igartua E, Contreras-Moreira B, Gracia MP, Casas AM. *HvFT1* polymorphism and effect-survey of barley germplasm and expression analysis. Frontiers in Plant Science. 2014;5 10.3389/fpls.2014.00251 PMC4047512 24936204

[40] Cockram J, Jones H, Leigh FJ, O'Sullivan D, Powell W, Laurie DA, et al Control of flowering time in temperate cereals: genes, domestication, and sustainable productivity. Journal of Experimental Botany. 2007;58: 1231–1244. 10.1093/jxb/erm042 17420173

[41] Wang G, Schmalenbach I, Korff MV, Léon J, Kilian B, Rode J, et al Association of barley photoperiod and vernalization genes with QTLs for flowering time and agronomic traits in a BC2DH population and a set of wild barley introgression lines. Theoretical and Applied Genetics. 2010;120: 1559–1574. 10.1007/s00122-010-1276-y 20155245 PMC2859222

[42] Hoffmann A, Maurer A, Pillen K. Detection of nitrogen deficiency QTL in juvenile wild barley introgression lines growing in a hydroponic system. BMC Genetics. 2012;13: 88 10.1186/1471-2156-13-88 23083378 PMC3584942

[43] Yan L, Loukoianov A, Tranquilli G, Helguera M, Fahima T, Dubcovsky J. Positional cloning of the wheat vernalization gene *VRN1*. Proceedings of the National Academy of Sciences. 2003;100: 6263–6268. 10.1073/pnas.0937399100 PMC156360 12730378

[44] von Korff M, Wang H, Léon J, Pillen K. AB-QTL analysis in spring barley: II. Detection of favourable exotic alleles for agronomic traits introgressed from wild barley (*H*. *vulgare ssp*. *spontaneum*). Theoretical and Applied Genetics. 2006;112: 1221–1231. 10.1007/s00122-006-0223-4 16477429

