# Correction: Genetic Mapping Reveals Broader Role of *Vrn-H3* Gene in Root and Shoot Development beyond Heading in Barley

**DOI:** 10.1371/journal.pone.0177612

**Published:** 2017-05-09

**Authors:** Md. Arifuzzaman, Süleyman Günal, Annemarie Bungartz, Shumaila Muzammil, Nazanin P. Afsharyan, Jens Léon, Ali Ahmad Naz

There are errors in the Funding section. The correct funding information is as follows: DAAD (German Academic Exchange Service) to Mr. Arifuzzaman. Uni-Bonn to Ali Ahmad Naz and German Research Foundation (DFG) to Süleyman Günal.

There is an omission in the final sentence of the Acknowledgements section. The sentence should read: German academic exchange service (DAAD) and German Research Foundation (DFG) funded PhD fellowships for this research work.

## References

[1] Arifuzzaman M, Günal S, Bungartz A, Muzammil S, Afsharyan NP., Léon J, et al (2016) Genetic Mapping Reveals Broader Role of *Vrn-H3* Gene in Root and Shoot Development beyond Heading in Barley. PLoS ONE 11(7): e0158718 https://doi.org/10.1371/journal.pone.0158718 27442506 10.1371/journal.pone.0158718 PMC4956229

