# Supplementary material for: Genetic Mapping Reveals Broader Role of Vrn-H3 Gene in Root and Shoot Development beyond Heading in Barley
Source: PLoS One. 2016 Jul 21;11(7):e0158718. doi: 10.1371/journal.pone.0158718 (PMC4956229; doi:10.1371/journal.pone.0158718)
Supplement: S1 File — Figure A, The alignment of Vrn-H3 genes between Strider, Triumph, Cheri and ICB181160 with MAFFT version 7. SNPs are with grey background. Figure B, Genetic interaction plot of Vrn-H2 and Vrn-H3 alleles from Cheri and ICB181160 for RDW (root dry weight). All nine possible allele combinations of both genes are plotted using R-interaction plot. Figure C, Genetic interaction plot of Vrn-H1 and Vrn-H3 alleles for RV (root volume). All nine possible allele combinations of both genes are plotted using R-interaction plot. Figure D, Genetic interaction plot of Vrn-H2 and Vrn-H3 alleles for RV (root volume). All nine possible allele combinations of both genes are plotted using R-interaction plot. Figure E, Genetic interaction plot of Vrn-H2 and Vrn-H3 alleles for RL (root length). All nine possible allele combinations of both genes are plotted using R-interaction plot. Figure F, Genetic interaction plot of Vrn-H2 and Vrn-H3 alleles for TIL (no. of tillers). All nine possible allele combinations of both genes are plotted using R-interaction plot. Table A, List of primers used for the genotyping and sequencing of the candidate genes. Forward (F) and reverse orientation (R) primers. Table B, Barley haplotype scoring from five non-synonymous SNPs and one SSR (simple sequence repeat) in the exon1 and exon 2 of ZCCT-Ha gene. Table C, Barley haplotype scoring from six non synonymous SNPs in the exon1 and exon 2 of ZCCT-Hb gene. Table D, Barley haplotype scoring from two described SNPs in the exon8 of PPDH1 gene. Table E, Barley haplotype scoring from two SNPs in the intron 3 of PPDH2 gene. Table F, Barley haplotype scoring from nine SNPs and three indels in the exon 2 and intron 2 of HvELF3 gene. Text A, Description of HvCCA1, HvLUX1 and HvCEN alleles in cultivar Cheri and ICB181160. (PDF) [file pone.0158718.s001.pdf]

Supplementary Information

Table A. List of primers used for the genotyping and sequencing candidate genes.

Candidate genes	Primer names	Primer sequences (5'–3')	PCR fragment size(bp)	Method	References
**VRN-H3**	HvVRNH3CAPS.F	CCGGGATATCTGTCACCAAC	349	Genotyping (CAPS : BclI)	This study
	HvVRNH3CAPS.R	CTTTCTTTTCAATTCGTTCCAG			This study
	VRNH3.F	TTGCTCCCTCATACCCTAGC	1170	Sequencing	This study
	VRNH3.R	CGTTGGATAATTTGGTGACTTGG			This study
**VRN-H1**	HvVRNH1.F	CCCAATATTTAGGCCTGACGG	550 (ICB), 400 (Cheri)	Genotyping (Indel)	This study
	HvVRNH1.R	CCTCACGATCCAGACCCAG			This study
**VRN-H2**	VRNH2.01F	ATGATGTCGCCCGTTCTT	235 (ICB), null (Cheri)	Genotyping	This study
	VRNH2.01R	GCCTGCGTCCACCTC			This study
	VRNH2.02F	ACCAGACCACACCAGAAACA	334 (ICB), null (Cheri)	Genotyping and sequencing	This study
	VRNH2.01R	GCCTGCGTCCACCTC			This study
	VRNH2a.R	CCTTTTGAAATAGTCAAACCAAGA	1803 (ICB), null (Cheri)		This study
	VRNH2b.R	CTCCTCCAATTGGTCAATCA	1778 (ICB), null (Cheri)		This study
**PPDH1**	PPDH1.F	CAACACCAATAACGGGAGCA	485	Sequencing	This study
	PPDH1.R	CCAGGAGATGAGACGAGCT			This study
**PPDH2**	FT3.01F	GTCTGTAGAGAGCTAGGTTGAC	1500	Sequencing	[8]
	FT3.02R	GTGATGCAACATTACAGTACAGC			[8]
**ELF3**	ELF3.01F	TGTCAGAGAAAGGCCTAAGAGA	170	Genotyping and sequencing	This study
	ELF3.01R	TGCTTTCCAGAAATGCTTTGGA			This study
	ELF3.02R	GCTCAAACACTTGGACAGCA	1020		[8]
**CCA1**	CCA1.F	ACCAGTTGTCAGCACTTCCT	556	Sequencing	This study
	CCA1.R	ATGGCTTCTGATTTGCACGG			This study
**LUX1**	LUX1.F	GCTCGATTGGTGTTGCTAGG	947	Sequencing	This study
	LUX1.R	GAGCAGAGAGCAGAGCATCC			This study
**CEN**	CEN.F	TCCTCTCATCTCCAGCCATC	999	Sequencing	This study
	CEN.R	TGCACGTACACTGGTTCACA			This study

Vrn-H3

```
                          -------------------------EXON1------------------------------
Strider   vrn-H3          atggccgggagggacagggatccgctgg-ttgtcggcagggttgtgggggacgtgctgga
Triumph   VRN-H3          atggccgggagggacagggatccgctgg-ttgtcggcagggttgtgggggacgtgctgga
Cheri     VRN-H3          --------agggacagggaatgcgctggattgtcggcagggttgtgggggacgtgctgga
ICB181160 vrn-H3          atggccgggagggacagggatccgctgg-ttgtcggcagggttgtgggggacgtgctgga
                                  ..**.  .**.** ****** *******************************

                          -------------------------EXON1------------------------------
Strider   vrn-H3          ccccttcgtccgaaccaccaacctcagggtgaccttcgggaacagggccgtgtccaacgg
Triumph   VRN-H3          ccccttcgtccgaaccaccaacctcagggtgaccttcgggaacagggccgtgtccaacgg
Cheri     VRN-H3          ccccttcgtccgaaccaccaacctcagggtgaccttcgggaacagggccgtgtccaacgg
ICB181160 vrn-H3          ccccttcgtccgaaccaccaacctcagggtgaccttcgggaacagggccgtgtccaacgg
                          ************************************************************

                          -------------------------EXON1------------------------------
Strider   vrn-H3          ctgcgagctcaagccgtccatggtcgcccagcagccgagggtggaggtgggcggcaatga
Triumph   VRN-H3          ctgcgagctcaagccgtccatggtcgcccagcagccgagggtggaggtgggcggcaatga
Cheri     VRN-H3          ctgcgagctcaagccgtccatggtcgcccagcagccgagggtggaggtgggcggcaatga
ICB181160 vrn-H3          ctgcgagctcaagccgtccatggtcgcccagcagccgagggtggaggtgggcggcaatga
                          ************************************************************

                          ---------EXON1--------------------------INTRON1-------------
Strider   vrn-H3          gatgaggaccttctacacgctcgtacgtacacaatcaccactatctaatgcaaattttaa
Triumph   VRN-H3          gatgaggaccttctacacgctcgtacgtacacaatcaccactatctaatgcaaattttaa
Cheri     VRN-H3          gatgaggaccttctacacgctcgtacgtacacaatcaccactatctaatgcaaattttaa
ICB181160 vrn-H3          gatgaggaccttctacacgctcgtacgtacacaatcaccactatctaatgcaaattttaa
                          ************************************************************

                          -----------------------63-INTRON1 ------80------------------
Strider   vrn-H3          tttagctctgaaactgaaagtgctcactacacgcacatgcacgatcgggctctctctagc
Triumph   VRN-H3          tttagctctgaaactgaaagtgcttaccacacgcacatgcacgatcgggctctctctagc
Cheri     VRN-H3          tttagctctgaaactgaaagtgctcaccacacgcacatgcacgatcgggctctctctagc
ICB181160 vrn-H3          tttagctctgaaactgaaagtgctcaccacacgcacatgcatgatcgggctctctctagc
                          ************************.**.*************.******************

                          -------------------------INTRON1----------------------------
Strider   vrn-H3          tagtacgtacgtccctttcgatgcttcttctctattcgatggaaattgatttcgatgc
Triumph   VRN-H3          tagtacgtacgtccctttcgatgcttcttctctattcgatggaaattgatttcgatgc
Cheri     VRN-H3          tagtacgtacgtccctttcgatgcttcttctctattcgatggaaattgatttcgatgc
ICB181160 vrn-H3          tagtacgtacgtccctttcgatgcttcttctctattcgatggaaattgatttcgatgc
                          ************************************************************

                          -------------------------INTRON1----------------------------
Strider   vrn-H3          ttctgttcatcacgtgtaatttgtgttaattagcaagcacatgattttctacgtaccatt
Triumph   VRN-H3          ttctgttcatcacgtgtaatttgtgttaattagcaagcacatgattttctacgtaccatt
Cheri     VRN-H3          ttctgttcatcacgtgtaatttgtgttaattagcaagcacatgattttctacgtaccatt
ICB181160 vrn-H3          ttctgttcatcacgtgtaatttgtgttaattagcaagcacatgattttctacgtaccatt
                          ************************************************************

                          -------------------------INTRON1 ---------------270-------
Strider   vrn-H3          tttctgtgctctctggtattattgatcatgattttctatgctttctttcaattcgttc
Triumph   VRN-H3          tttctgtgctctctggtattattgatcatgattttctatgctttctttctattcgttc
Cheri     VRN-H3          tttctgtgctctctggtattattgatcatgattttctatgctttctttctattcgttc
ICB181160 vrn-H3          tttctgtgctctctggtattattgatcatgattttctatgctttctttcaattcgttc
                          ***********************************************************

                          ---------------------302--INTRON1 ------------326----------
Strider   vrn-H3          caggaacattaatcatatataggagcttctgcaatcacatgactaatgctcgctcgatct
Triumph   VRN-H3          caggaacattaatcatatataggagcttctgcaatcacatgactaatgctcgctcgatct
Cheri     VRN-H3          caggaacattaatcatatataggagcttctgcaatcacatgactaatgctcgctcgatct
ICB181160 vrn-H3          caggaacattaatcatatataggtgcttctgcaatcacatgactaat----gctcgatct
                          ********************************************************

                          -------------------------INTRON1 ----------384-------------
Strider   vrn-H3          ttacatatatgcttatcagcttatgtacgtactctcgctggtgctgatcatgatatgtgc
Triumph   VRN-H3          ttacatatatgcttatcagcttatgtacgtactctcgctggtgctcatcatgatatgtgc
Cheri     VRN-H3          ttacatatatgcttatcagcttatgtacgtactctcgctggtgctcatcatgatatgtgc
ICB181160 vrn-H3          ttacatatatgcttatcagcttatgtacgtactctcgctggtgctgatcatgatatgtgc
                          ***********************************************************

                          -------INTRON1-------------------------EXON2 -------------36---
Strider   vrn-H3          atgctttatactgcctgcaggtgatggtagacccagatgctccaagtcctagcgacccca
Triumph   VRN-H3          atgctttatactgcctgcaggtgatggtagacccagatgctccaagtcctagcgacccca
Cheri     VRN-H3          atgctttatactgcctgcaggtgatggtagacccagatgctccaagtcctagcgacccca
ICB181160 vrn-H3          atgctttatactgcctgcaggtgatggtagacccagatgctccaagtcctagcgatccca
                          *******************************************************.****
```

```
                        --------EXON2----------------------INTRON2------------------
Strider   vrn-H3   accttagagagtatctccactggtaagtacagtaattgttactcagttgaatgatttctt
Triumph   VRN-H3   accttagagagtatctccactggtaagtacagtaattgttactcagttgaatgatttctt
Cheri    VRN-H3    accttagagagtatctccactggtaagtacagtaattgttactcagttgaatgatttctt
ICB181160 vrn-H3   accttagagagtatctccactggtaagtacagtaattgttactcagttgaatgatttc-t
                   ********************************************************** *

                   --------------------------INTRON2---------------------------
Strider   vrn-H3   tttccctagatcgatatacactagcggactagctca------tgtgtgtgtgtgtgtgta
Triumph   VRN-H3   tttccctagatcgatatacactagcggactagctcatgtgtgtgtgtgtgtgtgtgta
Cheri    VRN-H3    tttccctagatcgatatacactagcggactagctca------tgtgtgtgtgtgtgtgta
ICB181160 vrn-H3   tttccctagatcgatatacactagcagactagctca--tgtgtgtgtgtgtgtgtgta
                   *************************.**********      ******************

                   ----INTRON2-------------------------EXON3--------------------
Strider   vrn-H3   cgtacatgtgtgtgcaggttggtgacagatatcccgggtacaactggggcgtcgttcggg
Triumph   VRN-H3   cgtacatgtgtgtgcaggttggtgacagatatcccgggtacaactggggcgtcgttcggg
Cheri    VRN-H3    cgtacatgtgtgtgcaggttggtgacagatatcccgggtacaactggggcgtcgttcggg
ICB181160 vrn-H3   cgtacatgtgtgtgcaggttggtgacagatatcccgggtacaactggggcgtcgttcggg
                   ************************************************************

                   ---------------------------EXON3----------------------------
Strider   vrn-H3   caggaggtgatgtgctacgagagccctcgtccaaccatggggatccaccgcttcgtgctc
Triumph   VRN-H3   caggaggtgatgtgctacgagagccctcgtccaaccatggggatccaccgcttcgtgctc
Cheri    VRN-H3    caggaggtgatgtgctacg-----------------------------------------
ICB181160 vrn-H3   caggaggtgatgtgctacgagagccctcgtccaaccatggggatccaccgcttcgtgctc
                   *******************

                   ---------------------------EXON3----------------------------
Strider   vrn-H3   gtgctcttccagcagctggggcggcagacggtgtacgcccccgggtggcgccagaacttc
Triumph   VRN-H3   gtgctcttccagcagctggggcggcagacggtgtacgcccccgggtggcgccagaacttc
Cheri     VRN-H3   ------------------------------------------------------------
ICB181160 vrn-H3   gtgctcttccagcagctggggcggcagacggtgtacgcccccgggtggcgccagaacttc

                   ---------------------------EXON3----------------------------
Strider   vrn-H3   aacaccagggactttgccgagctctacaacctcggccagcccgttgccgccgtctacttc
Triumph   VRN-H3   aacaccagggactttgccgagctctacaacctcggccagcccgttgccgccgtctacttc
Cheri     VRN-H3   ------------------------------------------------------------
ICB181160 vrn-H3   aacaccagggactttgccgagctctacaacctcggccagccc------------------
```

Figure A. The alignment of *Vrn-H3* genes between Strider, Triumph, Cheri and ICB181160 with MAFFT version 7. SNPs are with grey background.

Vrn-H2

Table B. Barley haplotype scoring from five non-synonymous SNPs and one SSR(simple sequence repeat) in the exon 1 and exon 2 of *ZCCT-Ha* gene

**Cultivar/accession**	**Position of SSR and SNPs in Exon 1**					**Exon 2**	**Allele** [a] [1]	**Accession number**
	**127**[a]	**151**	**194**	**251**	**291**	**25**		
**Calicuchima-sib**	CAC (7)	A	G	G	C	A	*Vrn-H2*	*DQ492695*
**OWB-D**	CAC (5)	G	G	G	C	A	*Vrn-H2*	*DQ492697*
**Kompolti korai**	CAC (6)	G	T	A	C	A	*Vrn-H2*	*DQ492699*
**Dairokkaku**	CAC (5)	G	G	G	C	G	*Vrn-H2*	*AY485977*
**ICB181160**	CAC (7)	G	G	G	G	A	*Vrn-H2*	*This study*
**Cheri**	-	-	-	-	-	-	*vrn-H2*	*This study*

OWB-D : Oregon Wolfe Barley Dominant

[a]Number of CAC repeat (coding Histidine)

[b] Detection of presence/absence of the genes *ZCCT-Ha* and *ZCCT-Hb* after sequencing.

Table C. Barley haplotype scoring from six nonsynonymous SNPs in the exon1 and exon 2 of *ZCCT-Hb* gene

**Cultivar/accession**	**in Exon 1**		**in Exon 2**				**Allele** [a] [1]	**Accession number**
	**221**	**238**	**10**	**29**	**77**	**274**		
**Calicuchima-sib**	G	C	C	A	T	C	*Vrn-H2*	*DQ492696*
**OWB-D**	T	C	C	A	C	C	*Vrn-H2*	*DQ492698*
**Kompolti korai**	T	C	C	A	C	C	*Vrn-H2*	*DQ492700*
**Dairokkaku**	T	C	C	A	C	C	*Vrn-H2*	*AY485978*
**Caesarea 26-24**	T	T	A	G	T	G	*Vrn-H2*	*DQ492701*
**ICB181160**	T	T	C	G	T	G	*Vrn-H2*	*This study*
**Cheri**	-	-	-	-	-	-	*vrn-H2*	*This study*

OWB-D : Oregon Wolfe Barley Dominant

[a]Detection of presence/absence of the genes *ZCCT-Ha* and *ZCCT-Hb* after sequencing.

The genotyping of the *Vrn-H2* was performed with primers which were designed for detecting presence/absence of the *Vrn-H2* gene and furthermore for sequencing of zinc finger-CTT domain transcription factor a (*ZCCT-Ha*) and zinc finger-CTT domain transcription factor b (*ZCCT-Hb*). PCR results and sequencing were showed complete deletion of *Vrn-H2* gene in Cheri. Null allele of *vrn-H2* described as a recessive allele for spring growth habit in barley [1, 2]. Sequencing of *ZCC-Ha* (Table B) and *ZCC-Hb* (Table C) performed for only ICB181160 which has a dominant *Vrn-H2* allele and none of the designed primers amplified from Cheri because deletion of the complete gene.

Genetic interaction of *Vrn-H3* alleles with *Vrn-H1* and *Vrn-H2* alleles for root and shoot traits

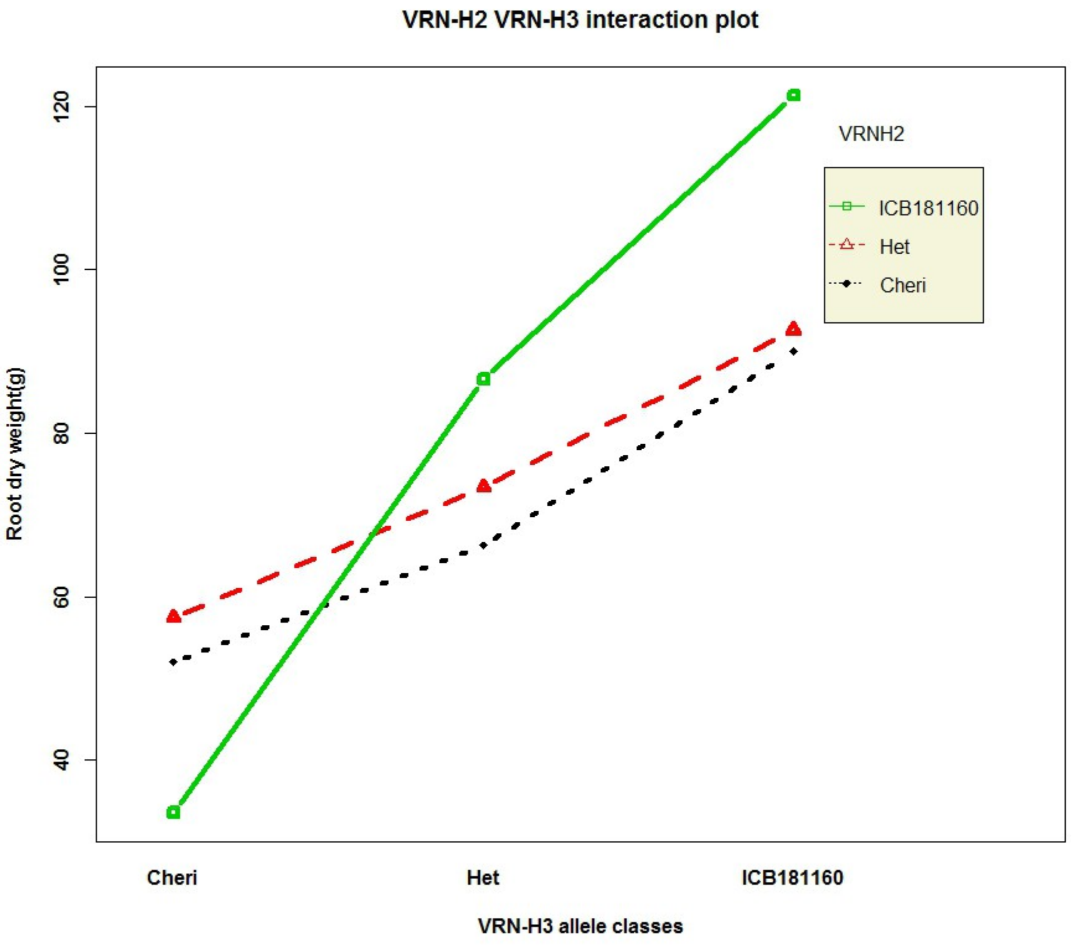

Figure B. Interaction plot of *Vrn-H2* and *Vrn-H3* alleles from Cheri and ICB181160 for RDW(root dry weight). All nine possible allele combinations are plotted using R-interaction plot.

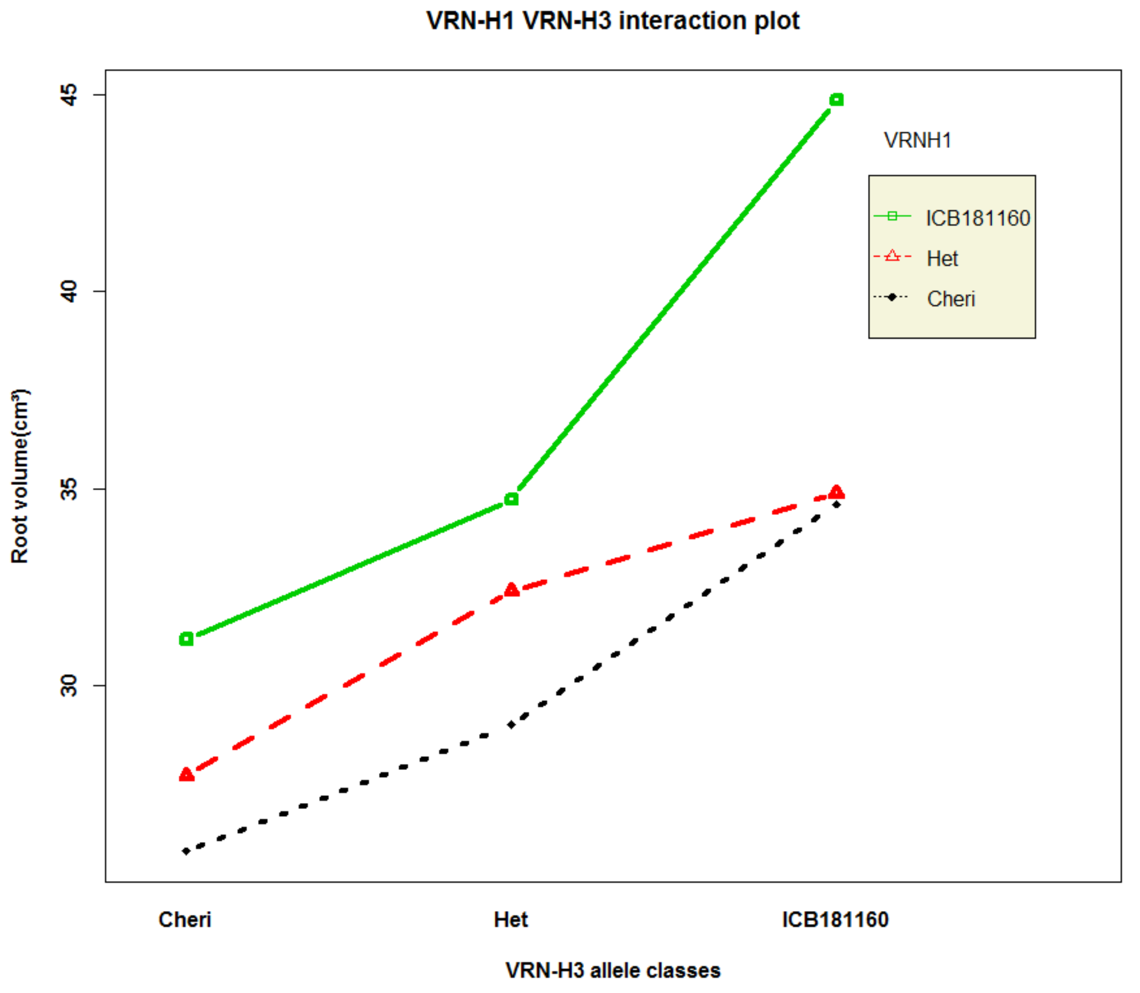

Figure C. Interaction plot of *Vrn-H1* and *Vrn-H3* alleles for RV(root volume). All nine possible allele combinations are plotted using R-interaction plot.

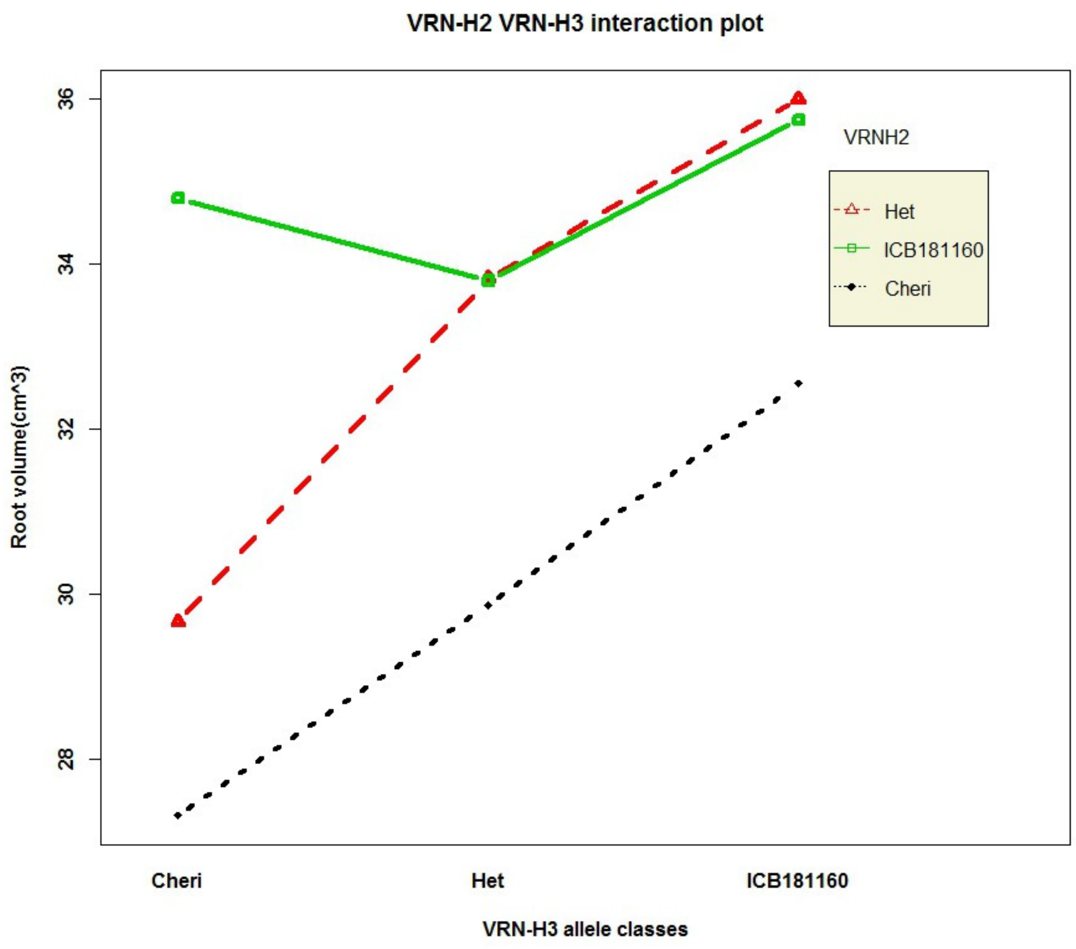

Figure D. Interaction plot of *Vrn-H2* and *Vrn-H3* alleles for RV (root volume). All nine possible allele combinations are plotted using R-interaction plot.

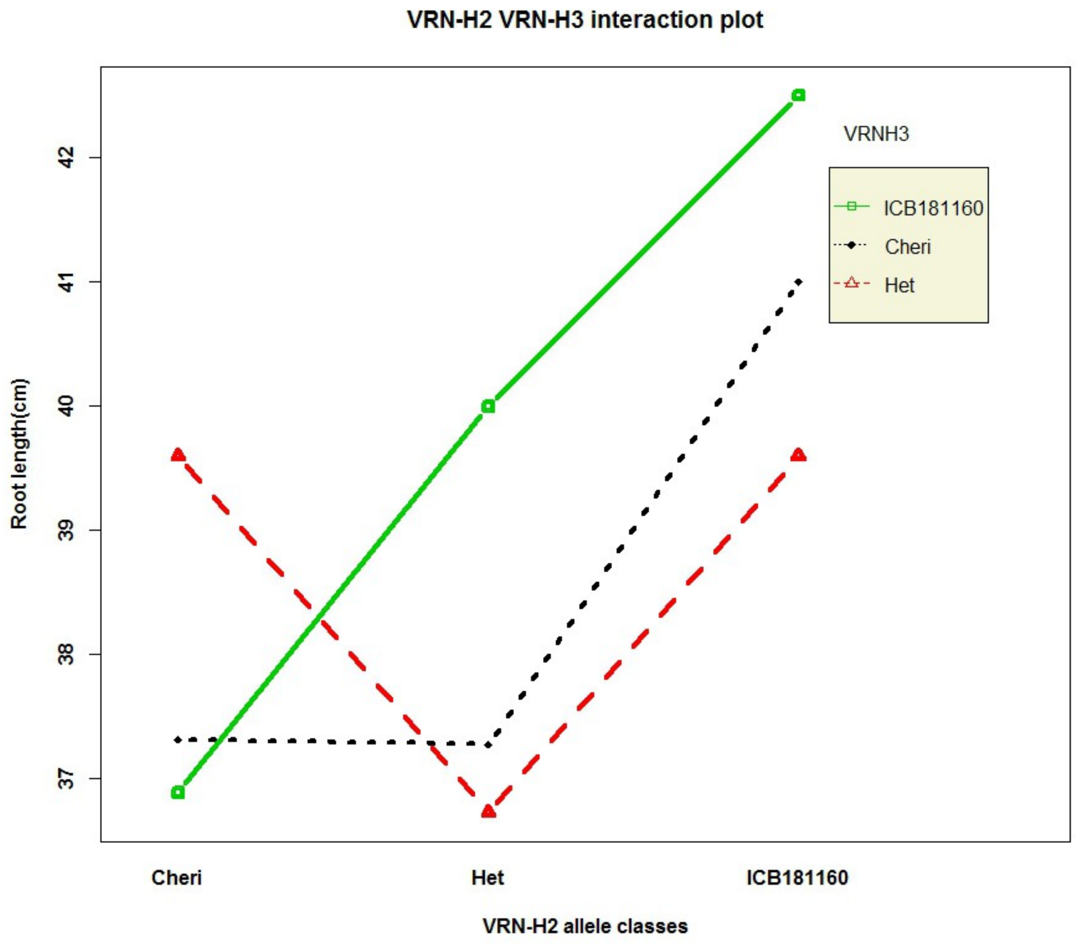

Figure E. Interaction plot of *Vrn-H2* and *Vrn-H3* alleles for RL (root length). All nine possible allele combinations are plotted using R-interaction plot.

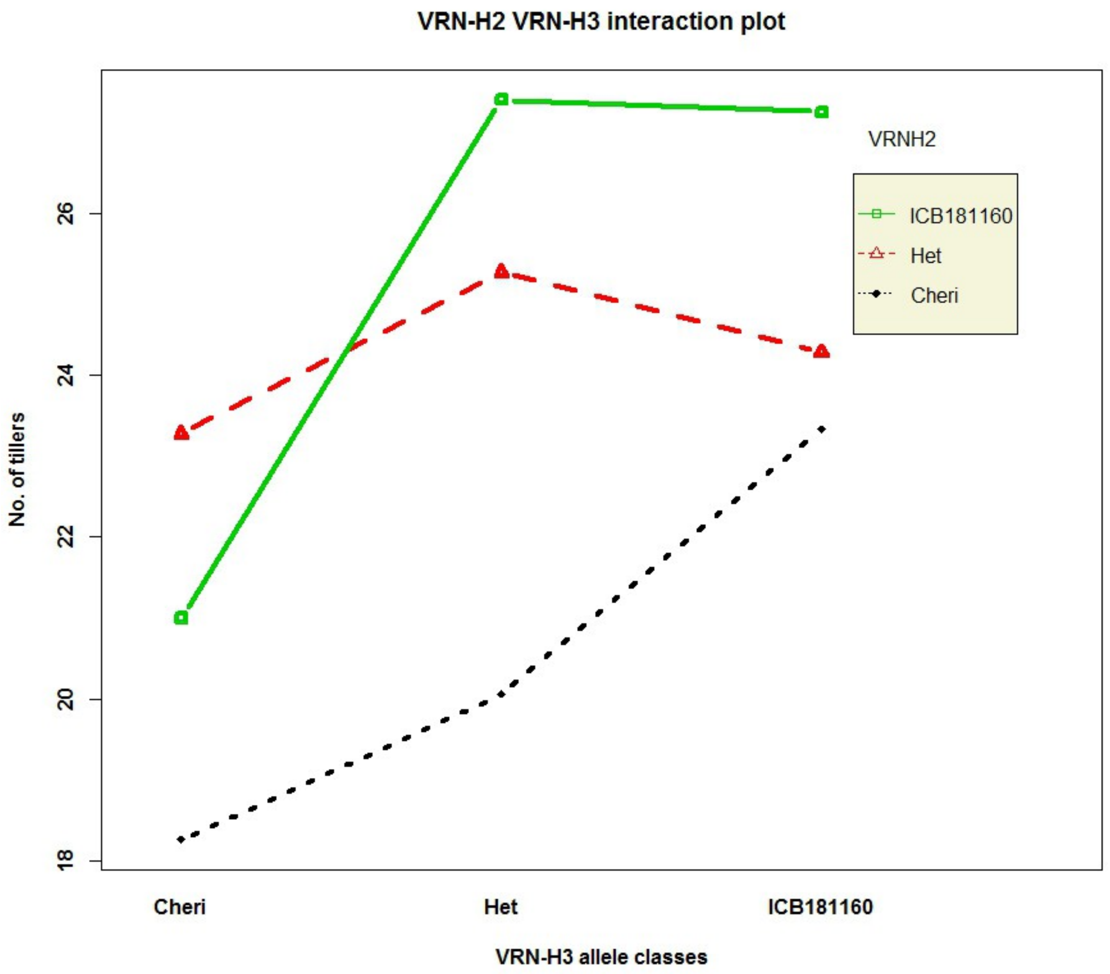

Figure F. Interaction plot of *Vrn-H2* and *Vrn-H3* alleles for TIL(no. of tillers). All nine possible allele combinations are plotted using R-interaction plot.

PPDH1

Table D. Barley haplotype scoring from two described SNPs in the exon8 of *PPDH1* gene

**Cultivar/accession**	**Position of polymorphisms** [a] [4]			**Accession number**
	**SNP22**	**SNP23**	**Allele**	
**Triumph**	T	A	*ppdh1*	*AY970704*
**Igri**	G	G	*PPDH1*	*AY970701*
**ICB181160**	G	G	*ppdh1*	*This study*
**Cheri**	T	A	*PPDH1*	*This study*

[a]Detection of the described SNPs in the exon 8 with sequencing.

The dominant *PPDH1* allele is prevalent in wild progenitor *H. vulgare spp. spontaneum* and accelerates flowering under long days in short growing seasons. The recessive *ppdh1* allele causes reduced response to long days and was selected for adaptation to long growing seasons [3]. Previous studies described polymorphisms between dominant and recessive *PPDH1* alleles. In this study, primers were designed spanning from exon 7 to end of exon 8for detection of previously described mutations(SNP22 and SNP23) [4]. Sequencing of *PPDH1* was showed that wild ICB181160 parent carrying a dominant *PPDH1* allele while Cheri carrying a recessive *ppdh1* allele.

PPDH2

Table E. Barley haplotype scoring from two SNPs in the intron 3 of *PPDH2* gene

**Cultivar/accession**	**Position of SNPin Intron 3**			**Accession number**
	**187**	**529**	**Allele** [a] [12]	
**Scarlett**	C	A	*PPDH2*	*EU331895*
**Morex**	C	G	*PPDH2*	*EU331873*
**Alexis**	C	A	*PPDH2*	*HM133570*
**OWB-D**	G	G	*PPDH2*	*EU331897*
**ICB181160**	G	G	*PPDH2*	*This study*
**Cheri**	C	A	*PPDH2*	*This study*

[a]Detection of the large deletion in the *PPDH2* with sequencing

The dominant *PPDH2* allele is functional and providing early flowering under short days whereas the recessive allele is truncated gene having only part of exon 4 and delay flowering under short days[5]. Previously shown that most of the spring cultivars carrying dominant allele of *PPDH2* gene whereas the majority of winter cultivars possessed recessive null *ppdh2*[6]. Sequencing of the *PPDH2* was carried out in Cheri and ICB181160. PCR results were showed that both of the parents carrying a dominant *PPDH2* allele. Two SNPs were detected in intron 3 which is previously described also by Casao et al. [7].

HvELF3

Table F: Barley haplotype scoring from nine SNPs and three indels in the exon 2 and intron 2 of *HvELF3* gene

**Cultivar/accession**	**Exon2**		**in Intron 2**										**Allele [d] [10]**	**Accession number**
	**751 [a]**	**852**	**179 [b]**	**235**	**242**	**332**	**334**	**377**	**465**	**573**	**705 [c]**	**721**		
**Bonus**	in	A	in	T	C	T	T	T	T	G	del	T	*Mat-a*	*JN180296*
**Igri**	in	G	del	C	T	C	C	C	A	C	in	C	*Mat-a*	*HQ850272*
**Mona**	del	A	in	T	C	T	T	T	T	G	del	T	*mat-a.8*	*HQ850275*
**ICB181160**	in	G	del	C	C	T	C	T	T	C	in	C	*EAM8*	*This study*
**Cheri**	in	G	del	C	T	C	C	C	A	C	in	C	*EAM8*	*This study*

[a] Letters “in” and “del” indicate a 4 bp indel (AAAG).

[b] Letters “in” and “del” indicate a 6 bp indel (TGCATT)

[c] Letters “in” and “del” indicate a 3 bp indel (AAG)

[d] Detection of large deletion and described mutations in exon 2 with sequencing

Transcript based cloning of the *EAM8* gene was showed that *EAM8* is the orthologue of the *Arabidopsis ELF3* and eam8 mutation has a dramatic effect on the expression level of circadian clock and photoperiod pathway genes [8]. First mutant line (*mat-a.8*) Mari was released commercially in 1961 as an early barley mutant [9]. In field trials under long-day and short-day conditions, it flowered earlier than the mother cultivar Bonus [9]. Primers were designed spanning from exon 2 to exon 3 for detection of previously described mutations [10] and sequencing of *HvELF3*. PCR results were showed that both of the parents carrying wild *EAM8* allele and do not have the detected mutations of *mat-a.8* or *ert-o.16*.

Text A. Description of *HvCCA1*, *HvLUX1* and *HvCEN* alleles in cultivar Cheri and ICB181160.

HvCCA1

A partially redundant *Arabidopsis thaliana* MYB-transcription factor *AtCCA1(CIRCADIAN CLOCK ASSOCIATED) 1* plays a role in the formation of morning active genes [13]. Previous study experimentally proofed that *HvCCA1* functionality similar to *AtCCA1* [11].Sequencing of the 556 bp genomic region spanning from exon1 to exon3 of *HvCCA1* sequenced from Cheri and ICB181160.Alignment of the sequences with available *HvCCA1* genes from other cultivars Morex, Triumph and Igri also were not showed any polymorphism. Sequencing results were showed that both of the parents having same allele of the *HvCCA1* gene.

HvLUX1

HvLUX1 is a candidate gene underlying the early maturity 10 (*eam10*) locus in barley. *HvLUX1* characterized as a transcription factor which encodes an MYB domain-containing SHAQKYF-type GARP family protein. The mutation identified in eam10 changed the first amino acid residue in this conserved motif and this described as *hvlux1* allele[3]. Sequencing of the complete gene was carried out and results were showed that Cheri and ICB181160 both carrying haplotype 1 allele of *HvLUX1* gene.

HvCEN

HvCEN (CENTRORADIALIS) is a barley homologue of Arabidopsis *TFL1(TERMINAL FLOWER1)*, which regulates inflorescence architecture and flowering time [15]. Three major haplotypes (I, II and III) identified at higher frequencies in domesticated barleys compare to wild progenitors. Major haplotypes I and III (which are later flowering than haplotype II in domesticated barleys) both harbor the mutation encoding the p.Pro135Ala alteration. The haplotype I is most frequently found in wild winter barleys from Fertile Crescent while haplotype III has been selected from wild barley and is completely fixed in northern European spring barley cultivars [15] . In this study *HvCEN* sequences of Cheri and ICB181160 were sequenced and aligned with previously described haplotypes, results were showed that Cheri is carrying a haplotype III allele of *HvCEN* gene while ICB181160 carrying a haplotype I.

REFERENCES

1. Yan L, Loukoianov A, Blechl A, Tranquilli G, Ramakrishna W, SanMiguel P et al. The wheat *VRN2* gene is a flowering repressor down-regulated by vernalization. Science. 2004;303: 1640–1644. doi:10.1126/science.1094305
2. Dubcovsky J, Chen C, Yan L. Molecular characterization of the allelic variation at the *VRN-H2* vernalization locus in barley. Molecular Breeding. 2005;15: 395–407. doi:10.1007/s11032-005-0084-6
3. Campoli C, Pankin A, Drosse B, Casao CM, Davis SJ, von Korff M. *HvLUX1* is a candidate gene underlying the early maturity 10 locus in barley: phylogeny, diversity, and interactions with the circadian clock and photoperiodic pathways. New Phytologist. 2013;199: 1045–1059. doi:10.1111/nph.12346
4. Turner A, Beales J, Faure S, Dunford RP, Laurie DA. The pseudo-response regulator *Ppd-H1* provides adaptation to photoperiod in barley. Science. 2005;310: 1031–1034. doi:10.1126/science.1117619
5. Kikuchi R, Kawahigashi H, Ando T, Tonooka T, Handa H. Molecular and functional characterization of PEBP genes in barley reveal the diversification of their roles in flowering. Plant Physiology. 2009;149: 1341–1353. doi:10.1104/pp.108.132134
6. Casao MC, Karsai I, Igartua E, Gracia MP, Veisz O, Casas AM. Adaptation of barley to mild winters: A role for *PPDH2*. BMC Plant Biology. 2011;11: 164. doi:10.1186/1471-2229-11-164
7. Casao MC, Igartua E, Karsai I, Lasa JM, Gracia MP, Casas AM. Expression analysis of vernalization and day-length response genes in barley (*Hordeum vulgare* L.) indicates that *VRNH2* is a repressor of *PPDH2 (HvFT3)* under long days. Journal of Experimental Botany. 2010;62: 1939–1949. doi:10.1093/jxb/erq382
8. Faure S, Turner AS, Gruszka D, Christodoulou V, Davis SJ, Korff MV, et al. Mutation at the circadian clock gene *EARLY MATURITY 8* adapts domesticated barley (*Hordeum vulgare*) to short growing seasons. Proceedings of the National Academy of Sciences. 2012;109: 8328–8333. doi:10.1073/pnas.1120496109
9. Dormling I, Gustafsson Å, Jung HR, von Wettstein D. Phytotron cultivation of Svalöf's Bonus barley and its mutant Svalöf's Mari. Hereditas. 1966;56:221–237. 10.1111/j.1601-5223.1966.tb02078.x

10 Zakhrabekova S, Gough SP, Braumann I, Muller AH, Lundqvist J, Ahmann K, et al. Induced mutations in circadian clock regulator *Mat-a* facilitated short-season adaptation and range extension in cultivated barley. Proceedings of the National Academy of Sciences. 2012;109: 4326–4331. doi:10.1073/pnas.1113009109

11 Kusakina J, Rutterford Z, Cotter S, Martí MC, Laurie DA, Greenland AJ, et al. Barley *Hv CIRCADIAN CLOCK ASSOCIATED* 1 and *Hv PHOTOPERIOD H1* are circadian regulators that can affect circadian rhythms in Arabidopsis. PLoS ONE. 2015;10. doi:10.1371/journal.pone.0127449

12 Hemming MN, Fieg S, Peacock WJ, Dennis ES, Trevaskis B. Regions associated with repression of the barley (*Hordeum vulgare*) *VERNALIZATION1* gene are not required for cold induction. Molecular Genetics and Genomics. 2009;282: 107–117. doi:10.1007/s00438-009-0449-3

13 Locke JCW, Kozma-Bognár L, Gould PD, Fehér B, Kevei É, Nagy F, et al. Experimental validation of a predicted feedback loop in the multi-oscillator clock of *Arabidopsis thaliana*. Molecular Systems Biology. 2006;2. doi:10.1038/msb4100102

14 Katoh K, Standley DM. MAFFT Multiple Sequence Alignment Software Version 7: Improvements in performance and usability. Molecular Biology and Evolution. 2013;30: 772–780. doi:10.1093/molbev/mst010.

15 Comadran J, Kilian B, Russell J, Ramsay L, Stein N, Ganal M, et al. Natural variation in a homolog of *Antirrhinum CENTRORADIALIS* contributed to spring growth habit and environmental adaptation in cultivated barley. Nature Genetics. 2012;44: 1388–1392. doi:10.1038/ng.2447
